# Unveiling superior phenol detoxification and degradation ability in *Candida tropicalis* SHC-03: a comparative study with *Saccharomyces cerevisiae* BY4742

**DOI:** 10.3389/fmicb.2024.1442235

**Published:** 2024-09-11

**Authors:** Qian Li, Yulei Chen, Hao Tang, Baichao Shu, Zhengyue Zhang, Jiaye Tang, Dang Li, Linjia Jiang, Jiwei Shen, Yaojun Yang, Hanyu Wang, Menggen Ma

**Affiliations:** ^1^College of Resources, Sichuan Agricultural University, Chengdu, China; ^2^Bamboo Diseases and Pests Control and Resources Development Key Laboratory of Sichuan Province, College of Life Science, Leshan Normal University, Leshan, China; ^3^Key Laboratory of Land Resources Evaluation and Monitoring in Southwest (Sichuan Normal University), Ministry of Education, Chengdu, China; ^4^Institute of Landscape Ecology, University of Münster, Münster, Germany

**Keywords:** *Candida tropicalis*, *Saccharomyces cerevisiae*, phenol, degradation, reactive oxygen species

## Abstract

This study examined the phenol degradation capabilities and oxidative stress responses of *Candida tropicalis* SHC-03, demonstrating its metabolic superiority and resilience compared to *Saccharomyces cerevisiae* BY4742 in a culture medium with phenol as the sole carbon source. Through comparative growth, transcriptomic, and metabolomic analyses under different phenol concentrations, this study revealed *C. tropicalis* SHC-03’s specialized adaptations for thriving in phenol as the sole carbon source environments. These include a strategic shift from carbohydrate metabolism to enhanced phenol degradation pathways, highlighted by the significant upregulation of genes for Phenol 2-monoxygenase and Catechol 1,2-dioxygenase. Despite phenol levels reaching 1.8 g/L, *C. tropicalis* exhibits a robust oxidative stress response, efficiently managing ROS through antioxidative pathways and the upregulation of genes for peroxisomal proteins like *PEX2*, *PEX13*, and *PMP34*. Concurrently, there was significant upregulation of genes associated with membrane components and transmembrane transporters, enhancing the cell’s capacity for substance exchange and signal transduction. Especially, when the phenol concentration was 1.6 g/L and 1.8 g/L, the degradation rates of *C. tropicalis* towards it were 99.47 and 95.91%, respectively. Conversely, *S. cerevisiae* BY4742 shows limited metabolic response, with pronounced growth inhibition and lack of phenol degradation. Therefore, our study not only sheds light on the molecular mechanisms underpinning phenol tolerance and degradation in *C. tropicalis* but also positions this yeast as a promising candidate for environmental and industrial processes aimed at mitigating phenol pollution.

## 1 Introduction

Phenol, a widely used industrial chemical in the pharmaceutical, resin synthesis, and plastics manufacturing sectors, is a prevalent contaminant in industrial effluents (Schmidt, 2005; Motamedi et al., 2022). Its high stability and toxicity necessitate effective and eco-friendly methods for its removal, essential for environmental protection (Guerra, 2001; Kujawski et al., 2004; Pinto et al., 2005). The adverse effects of phenol on aquatic life and human health, ranging from headaches and dizziness to severe respiratory, neurological, and cardiovascular issues, underscore the urgency for efficient remediation techniques (Panigrahy et al., 2022; Grace Pavithra et al., 2023). Biodegradation stands out as an eco-friendly and efficient approach to addressing phenol pollution. Microorganisms play a crucial role in this process, converting phenol into less toxic substances (Agarry et al., 2008; Gu et al., 2016). Although bacteria are commonly utilized for their rapid growth and adaptability, their efficiency and stability often diminish at high phenol concentrations or in complex environmental conditions (Gu et al., 2016; Li et al., 2019; Mahgoub et al., 2023).

Yeast, particularly *Saccharomyces cerevisiae* and *Candida tropicalis*, have long been subjects of intense study due to the distinct physiological and metabolic characteristics (Phalgune et al., 2013; Hegazy et al., 2024). Recent advances have highlighted their differential adaptation strategies to environmental stresses, including phenol and other phenolic compounds (Adav et al., 2007; Yang et al., 2012; He et al., 2022). *S. cerevisiae* has been studied for its resilience against phenolic compound concentrations (Tekarslan et al., 2015). These studies have demonstrated that *S. cerevisiae* combats phenolic stress by detoxifying harmful compounds through oxidation–reduction processes, enhancing cellular defenses against oxidative stress, maintaining membrane integrity, and ensuring energy production and cellular function through mitochondrial activities (Ding et al., 2012; Gu et al., 2019).

Additionally, *C. tropicalis* has been recognized for its remarkable phenol biodegradation capabilities, employing specific enzymatic pathways for converting phenol to carbon dioxide and water, even under high concentrations that typically exert inhibitory effects (Varma and Gaikwad, 2009; Phalgune et al., 2013). This efficiency is due to a unique genetic and metabolic framework, including the activation of genes encoding for phenol hydroxylase and other key enzymes involved in the catabolism of phenolic compounds, enabling *C. tropicalis* to excel in phenol biodegradation tasks (Adav et al., 2007; Phalgune et al., 2013; He et al., 2022). In our previous studies, we discovered that *C. tropicalis* exhibits high tolerance to phenol (Wang et al., 2020). Using a complete Yeast extract peptone dextrose (YPD) supplemented with phenol, we observed that *C. tropicalis* does not activate its phenol degradation mechanisms when other more readily degradable carbon sources (such as glucose) are present. This finding suggests that *C. tropicalis* possesses a regulatory mechanism that prioritizes the utilization of less toxic carbon sources over phenol under nutrient-rich conditions (Wang et al., 2020). Building on this foundation and guided by prior research, this study employs a medium where phenol is the sole carbon source. This experimental shift is crucial for unveiling the comprehensive adaptability of *C. tropicalis* to metabolize phenol in the absence of alternative carbon sources, offering insights into the yeast’s specific metabolic pathways and regulatory networks involved in phenol tolerance and degradation.

Therefore, this study seeks to investigate the specific pathways and regulatory mechanisms that enable *C. tropicalis* to utilize phenol as a sole carbon source, contrasting with the phenol response strategies of *S. cerevisiae*. This exploration aims to provide a comprehensive understanding of yeast adaptability and potential biotechnological applications in environmental remediation, highlighting the sophisticated metabolic engineering that underpins phenol degradation in yeasts.

## 2 Materials and methods

### 2.1 Strain and culture conditions

*C. tropicalis* SHC-03 was isolated from the distillery lees of Shehong Brewery in Sichuan Province, while *S. cerevisiae* BY4742 from Open Biosystems, Inc. (United States).

Yeast extract Peptone Dextrose (YPD) (Wang et al., 2020) Medium Preparation: YPD medium was prepared with the following composition per liter: 20 g domestic peptone, 10 g yeast extract powder, and 20 g glucose. The pH of the medium was adjusted to 6.0. For solid media, 20 g of agar per liter was added. The medium was sterilized by autoclaving at 121°C for 20 min. Glucose was autoclaved separately at 115°C for 20 min to avoid Maillard reactions that could occur at higher temperatures. All reagents used for the YPD medium were sourced from Chengdu Haoboyou Company.

Phenol-Only Carbon Source Medium Preparation (Yoneda et al., 2016): The medium for phenol utilization studies was composed of the following per liter: 0.4 g K_2_HPO_4_, 0.2 g KH_2_PO_4_, 0.1 g NaCl, 0.1 g MgSO_4_, 0.01 g MnSO_4_·H_2_O, 0.01 g Fe_2_(SO_4_)_3_·H_2_O, 0.01 g Na_2_MoO_4_·2H_2_O, and 0.4 g (NH_4_)_2_SO_4_. This medium was autoclaved at 121°C for 20 min. Phenol was added post-sterilization to achieve the desired concentration (If glucose was used in place of phenol, it served as the sole carbon source). Reagents for medium were obtained from Chengdu Haoboyou Company.

Other Reagents: 2′,7′-Dichlorofluorescein diacetate was procured from Sigma-Aldrich, and other reagents, including ammonia, NH_4_Cl, 4-aminoantipyrine, and K_3_(FeCN)_6_, were purchased from Chengdu Haoboyou Company.

### 2.2 Incubated strains and measured growth curves

*C. tropicalis* SHC-03 and *S. cerevisiae* BY4742 were inoculated into YPD liquid medium and incubated on a constant temperature shaker set at 30°C and 200 rpm overnight to cultivate the cells. Following incubation, the cell suspension was washed three times with sterile water to remove residual medium components. Subsequently, the cells were transferred into a medium containing phenol as the sole carbon source, prepared at varying concentrations, and incubated under identical conditions (30°C and 200 rpm). Growth was monitored by measuring the optical density at 600 nm (*OD_600_*) using an ultraviolet spectrophotometer at various time intervals. The *OD_600_* readings were used to determine cell concentration and construct growth curves for each strain across the different phenol concentrations.

### 2.3 Determination of phenol degradation rate

The residual concentration of phenol in the medium was quantified using the 4-aminoantipyrine spectrophotometric method. This method is based on the reaction between phenol, 4-aminoantipyrine, and potassium ferricyanide in an alkaline environment, resulting in the formation of a red complex. The intensity of the color, indicative of the phenol concentration, was measured by recording the absorbance at 510 nm (*OD_510_*) (Mosae Selvakumar, 2018).

### 2.4 Intracellular reactive oxygen species detection

Cells incubated overnight were washed thrice and incubated in a glucose-only carbon source medium for 3 h (cells collected at this point were labeled as 0 h). After another wash, cells were transferred to phenol-only carbon source medium which with 1.8 g/L phenol and cultured for 6 h at 30°C and 200 rpm, and cells collected at 6 h were labeled accordingly. Cells from both time points were adjusted to *OD_600_* = 1.0 in 1.5 mL Eppendorf tubes. The cell pellet obtained after centrifugation was stained with 2′,7′-DCFdiacetate (pre-thawed), and fluorescence microscopy (equipped with a GFP filter) was used for visualization. Statistical analysis was performed to evaluate ROS accumulation in cells.

### 2.5 Sample preparation for transcriptome and metabolome analyses

Primary cultures of strains SHC-03 and BY4742 were washed thrice and incubated in glucose-only carbon source medium for 3 h (samples collected at this stage were designated as 0 h for both transcriptome and metabolome analyses). The remaining cells were washed thrice with sterile water and cultured in phenol-only carbon source medium for 6 h according to the initial concentration *OD_600_* = 0.3. Then the cells were collected (labeled 6 h for transcriptome and metabolome analysis). For the reliability of experimental data, three biological replicates were prepared for transcriptomics, and six for metabolomics. The remaining culture was monitored every 6 h to assess growth and ensure accurate sampling.

### 2.6 Transcriptome analysis

Total RNA was extracted using the TRIzol reagent (Thermo Fisher, Catalog No. 15596018). The quantity and integrity of the RNA were evaluated using the RNA Nano 6,000 Assay Kit on the Bioanalyzer 2,100 system (Agilent Technologies, CA, United States). Gene expression levels were estimated using the FPKM (Fragments Per Kilobase of transcript per Million mapped reads) method. For differential expression analysis, the DESeq2 package in R was utilized. Significantly differentially expressed genes (DEGs) were identified with a threshold of |log2(foldchange)| ≥ 1 and an adjusted *p*-value (padj) ≤ 0.05. Enrichment analyses for KEGG pathways and GO terms of the DEGs were conducted using the DAVID tool.^1^

### 2.7 Metabolome analysis

Untargeted metabolomic analysis was performed using a Vanquish UHPLC system (Thermo Fisher, Germany) coupled to an Orbitrap Q Exactive™ HF mass spectrometer (Thermo Fisher, Germany). Raw data files underwent processing with Compound Discoverer 3.1 (CD3.1, Thermo Fisher) for peak alignment, picking, and quantitation of each metabolite. Statistical analyses of the metabolomic data were conducted using R and Python programming languages. Metabolites were annotated against the KEGG database and conducting enrichment analysis using the MetaboAnalyst tool^2^ to identify their metabolic pathways.

### 2.8 Integrated analysis of transcriptome and metabolome

The interaction between metabolites and genes was illustrated through network mapping, selecting the top 10 differentially expressed genes and top 5 differentially expressed metabolites for this purpose. Both sets of data were mapped against the KEGG pathway database to identify common pathways, focusing on major metabolic and signal transduction pathways. The iPath tool^3^ was used to visualize the enrichment pathways common to both differential metabolites and genes, providing a comprehensive overview of metabolic pathways enriched by both metabolomic and transcriptomic analyses.

## 3 Results and analysis

### 3.1 Effects of different phenol concentrations on cell growth and phenol degradation

**Figure 1:**
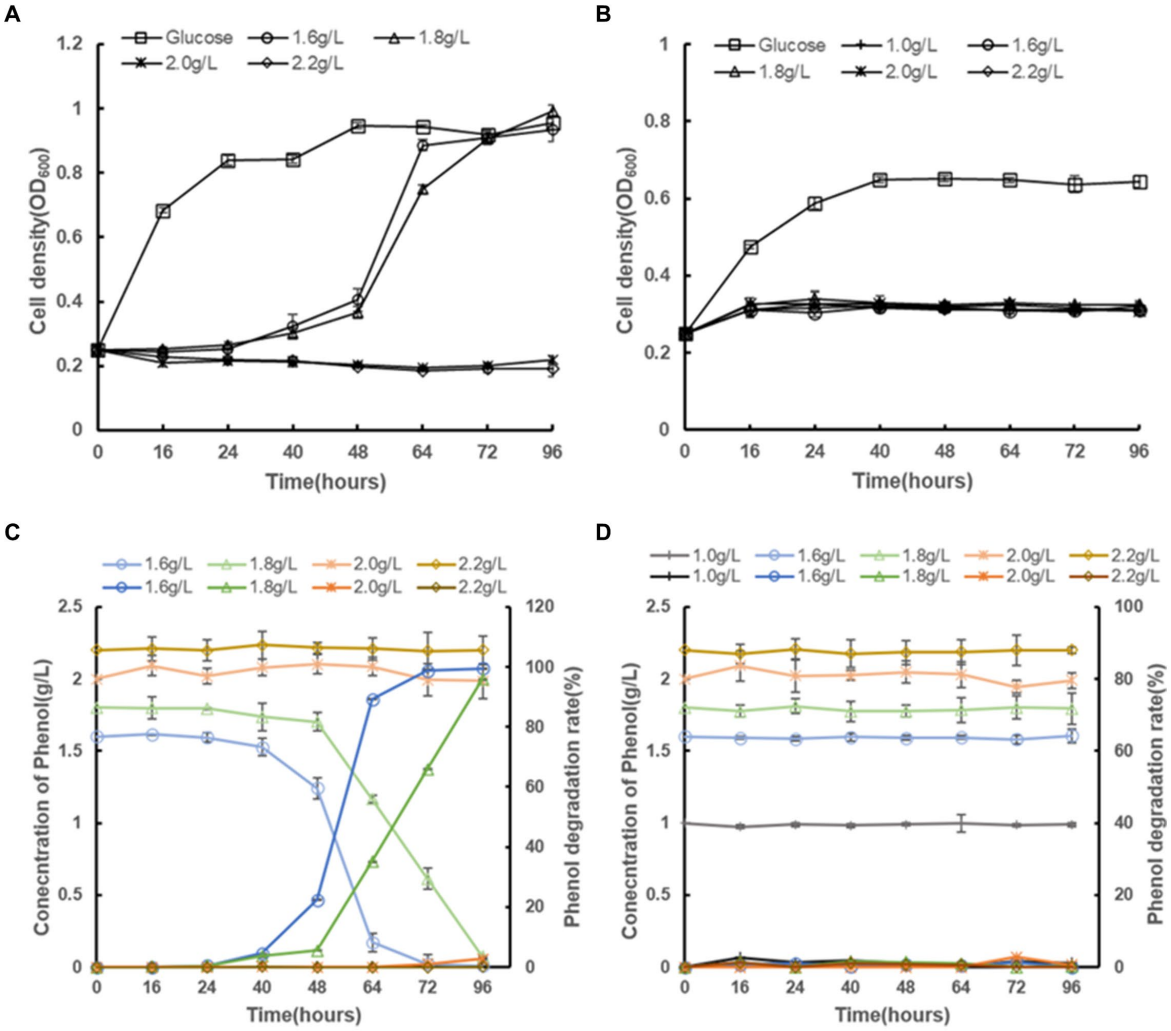
The growth profiles of *C. tropicalis* SHC-03 and *S. cerevisiae* BY4742 in different culture media, as well as the residual phenol concentrations and phenol degradation rates in the culture media. **(A)** Growth curve of *C. tropicalis* SHC-03. **(B)** Growth curve of *S. cerevisiae* BY4742. **(C)** Phenol concentration and phenol degradation rate in the culture medium (*C. tropicalis* SHC-03). **(D)** Phenol concentration and phenol degradation rate in the culture medium (*S. cerevisiae* BY4742).

This study investigated the impact of various phenol concentrations on the growth of *C. tropicalis* SHC-03, using two sets of culture conditions. One set used media supplemented with 20.0 g/L glucose as the sole carbon source to serve as a control group without inhibitors. The other set employed media where phenol was the sole carbon source, with phenol added at concentrations of 1.6 g/L, 1.8 g/L, 2.0 g/L, and 2.2 g/L, with no glucose included. *C. tropicalis* SHC-03 demonstrated normal growth in media with 20.0 g/L glucose (Figure 1A). However, growth was notably inhibited at phenol concentrations of 1.6 g/L and 1.8 g/L, with cellular growth resuming only after 24 h of incubation (Figure 1A). Notably, after 96 h of cultivation, phenol degradation rates were observed to reach 99.47 and 95.91%, respectively, for 1.6 g/L and 1.8 g/L concentrations (Figure 1C). Conversely, in media with 2.0 g/L and 2.2 g/L phenol, growth was entirely inhibited, with the phenol concentration in the culture medium remaining largely unchanged, suggesting a complete halt in cellular activity under these conditions (Figure 1C). So, a phenol concentration of 1.8 g/L was selected for subsequent experiments. This concentration effectively maintains the cells in a recoverable state of growth inhibition, allowing for further investigation into the degradation capabilities and adaptability of *C. tropicalis* SHC-03 under phenolic stress.

In parallel, *S. cerevisiae* BY4742 was subjected to the same conditions. While the strain BY4742 displayed normal growth in media where glucose served as the sole carbon source, it exhibited no growth in media supplemented with phenol, regardless of the concentration. This was accompanied by an unchanged phenol concentration within the culture medium, reinforcing the observation that *S. cerevisiae* lacks the capacity to utilize phenol as a carbon source under the tested conditions (Figures 1B,D).

### 3.2 Transcriptomic and metabolomic analysis under phenol stress conditions

Sequencing analysis revealed that both *C. tropicalis* SHC-03 and *S. cerevisiae* BY4742 undergo significant changes in gene expression upon phenol exposure. The sequencing quality metrics, with clean bases predominantly exceeding 6.0 G and both Q20 and Q30 above 90%, underscore the high fidelity of the data, setting a robust foundation for subsequent analyses (Supplementary Table S1). Correlation analysis between samples showed high consistency among biological replicates, further validating the reliability of the data for subsequent research analysis (Supplementary Figures S1, S2).

**Figure 2:**
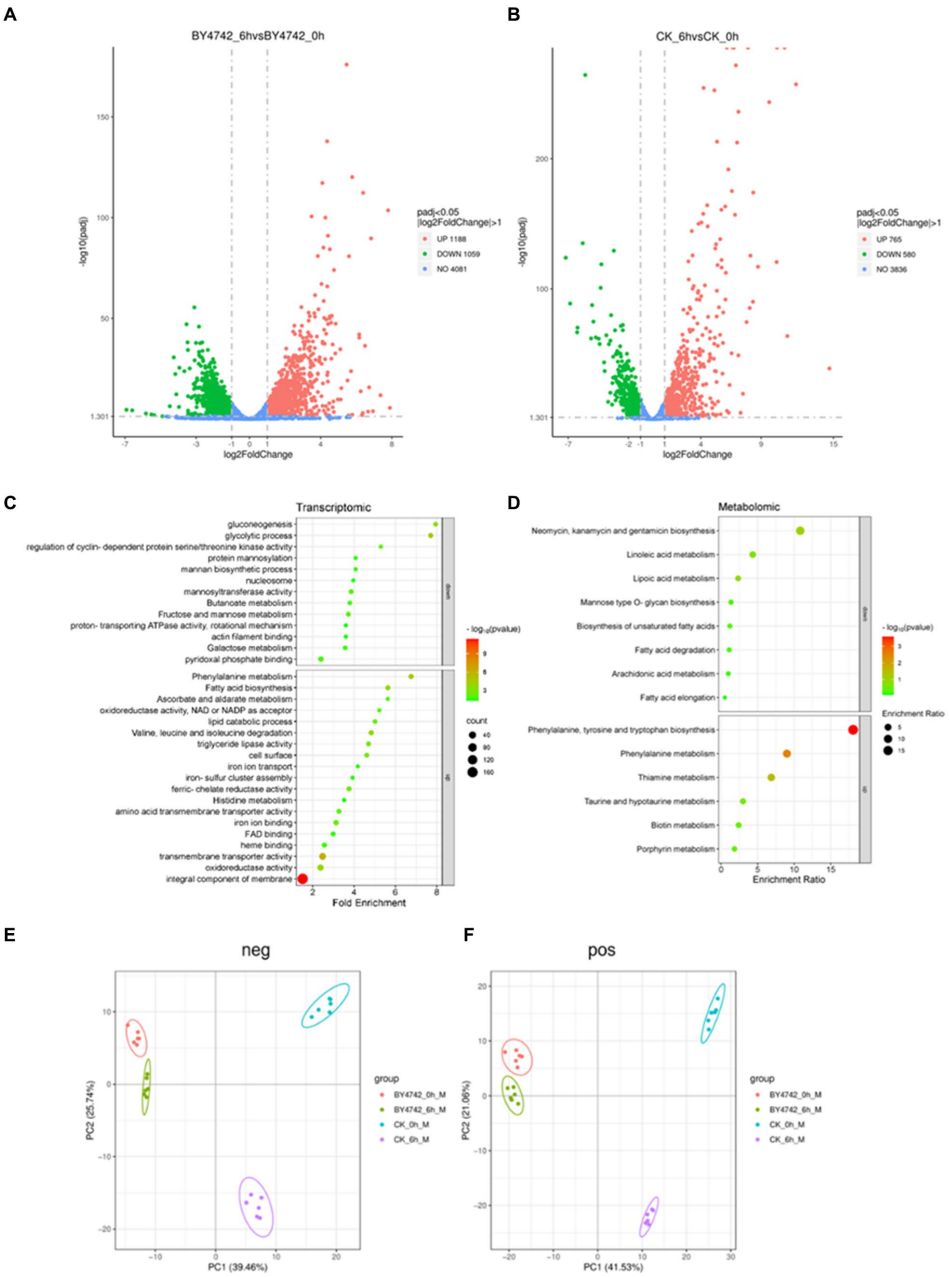
Transcriptome and metabolome analysis of *C. tropicalis* SHC-03 and *S. cerevisiae* BY4742. **(A)** Volcano plot of differentially expressed genes in *Saccharomyces cerevisiae* BY4742. **(B)** Volcano plot of differentially expressed genes in *C. tropicalis* SHC-03. **(C)** Transcriptionally specific upregulation and downregulation pathways in *C. tropicalis* SHC-03. **(D)** Metabolic pathways with specific upregulation and downregulation in *C. tropicalis* SHC-03. **(E)** Principal component analysis (PCA) plot of positive metabolites between different samples. **(F)** Principal component analysis (PCA) plot of negative metabolites between different samples (CK_0h: *C. tropicalis* SHC-03 0 h; CK_6h: *C. tropicalis* SHC-03 6 h).

Upon analyzing the sequencing data of *C. tropicalis* SHC-03 and *S. cerevisiae* BY4742, a notable change in gene expression was observed following phenol exposure. The comparison between pre-treatment (0 h) and post-treatment (6 h) revealed differential expression in both strains. Specifically, the strain SHC-03 displayed 1,345 differentially expressed genes, consisting of 765 upregulated and 580 downregulated genes. In contrast, the strain BY4742 exhibited a total of 2,247 differentially expressed genes, with 1,188 being upregulated and 1,059 downregulated. Volcano plots (Figures 2A,B) visually summarize these differential expressions, with further analyses for these genes.

Gene enrichment analyses, conducted via the DAVID tool. Upregulated genes of SHC-03 predominantly enriched pathways such as the “integral component of membrane” and “extracellular region,” whereas downregulated genes were mainly associated with pathways like “mitochondrion” and “plasma membrane” (Supplementary Tables S2, S3). For the strain BY4742, upregulated genes were enriched in pathways including “cellular response to heat” and “ribosome,” with downregulated genes enriched in “translation” and “cytoplasmic translation (Supplementary Tables S4, S5).” Specific upregulated pathways in the strain SHC-03 include “integral component of membrane,” “transmembrane transporter activity,” and “oxidoreductase activity,” among others, while specific downregulated pathways feature “nucleosome,” “glycolytic process,” and “gluconeogenesis,” to name a few (Figure 2C).

Pearson correlation analysis of metabolomic data showed a high correlation among replicate samples, validating the reliability of the dataset (Supplementary Figures S3, S4). Principal component analysis (PCA) highlighted distinct metabolomic profiles between strains SHC-03 and BY4742 post-phenol treatment, underscoring significant metabolic differences (Figures 2E,F). the strain SHC-03 experienced changes in 272 upregulated and 458 downregulated metabolites, while the strain BY4742 expressed 138 upregulated and 220 downregulated metabolites. MetaboAnalyst tool was utilized for enrichment analysis (Supplementary Figures S5–S8), with the strain SHC-03’s specific upregulated metabolites showed enrichment in “Phenylalanine, tyrosine and tryptophan biosynthesis,” and “Phenylalanine metabolism,” among others. Specific downregulated metabolites in the strain SHC-03 were enriched in pathways like “Fatty acid degradation (Figure 2D).”

In summary, *C. tropicalis* SHC-03 and *S. cerevisiae* BY4742 exhibit distinct transcriptomic and metabolomic responses under phenol stress, with the strain SHC-03 showed adaptations that may facilitate phenol degradation through specific metabolic pathways.

### 3.3 Activation of the oxidative stress response

**Figure 3:**
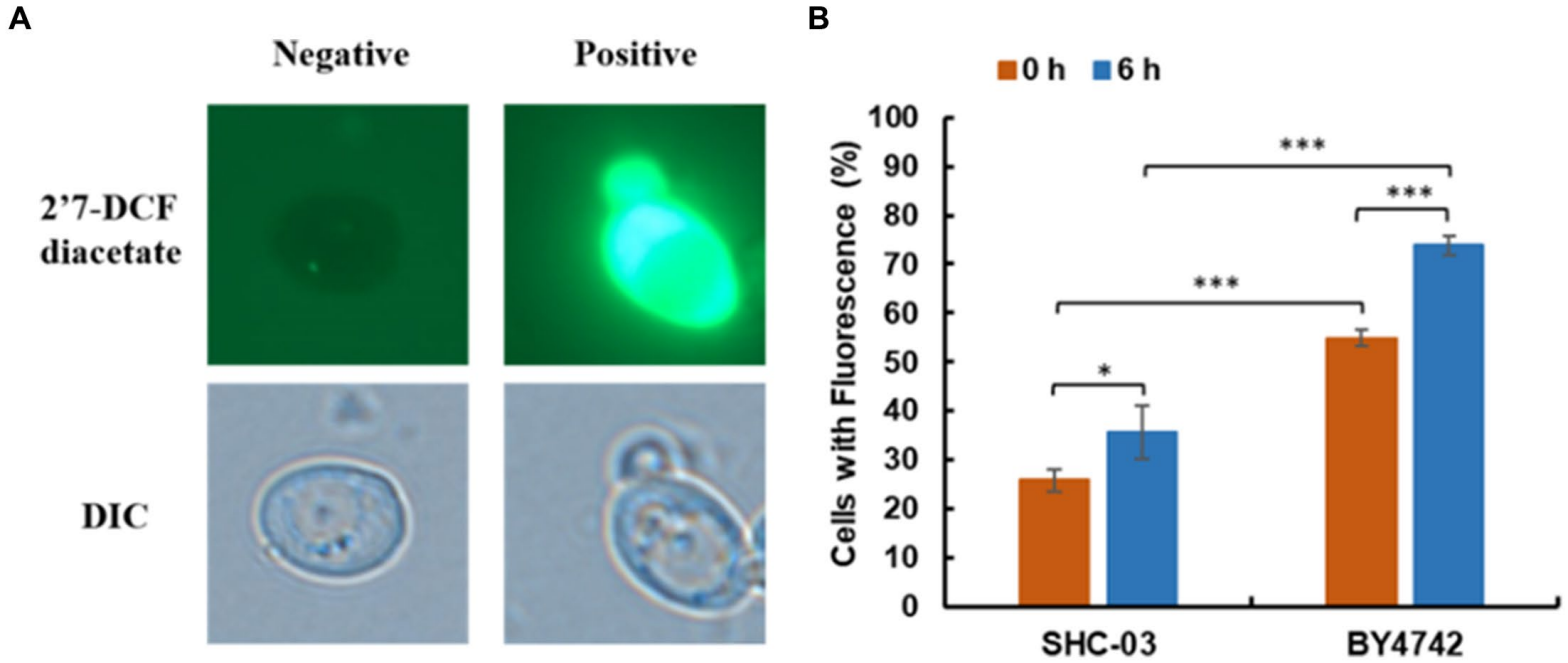
Reactive oxygen species accumulation in *C. tropicalis* SHC-03 and *S. cerevisiae* BY4742. **(A)** Accumulation of reactive oxygen species in cells. **(B)** The proportion of cells at 1.8 g/L phenol that contained reactive oxygen species after treatment for 0 and 6 h. 2′7′-DCF diacetate (top column): reactive oxygen species indicator dye. DIC (down column): differential interference microscope. Negative: no signal; Positive: there is a signal. * *p* < 0.05, *** *p* < 0.001 indicates significant differences. The data represent aver-ages of three experiments. At least 100 cells were examined on each bright-field image.

This investigation-stained cells of *C. tropicalis* SHC-03 and *S. cerevisiae* BY4742 to quantify the accumulation of reactive oxygen species (ROS). Under fluorescence microscopy, ROS-accumulating cells were distinguished by green fluorescence (Allen et al., 2010; Wang et al., 2023; Liao et al., 2024), while cells without ROS accumulation did not fluoresce (Figure 3A). Statistical analysis at 0 h revealed that 25.82% of *C. tropicalis* SHC-03 cells and 54.85% of *S. cerevisiae* BY4742 cells exhibited ROS accumulation. By 6 h, these figures rose to 35.65 and 73.87%, respectively, highlighting a significantly higher ROS accumulation in *S. cerevisiae* at both time points, and the proportion of cells accumulating ROS in the strain BY4742 was significantly higher than that in the strain SHC-03 (Figure 3B).

**Figure 4:**
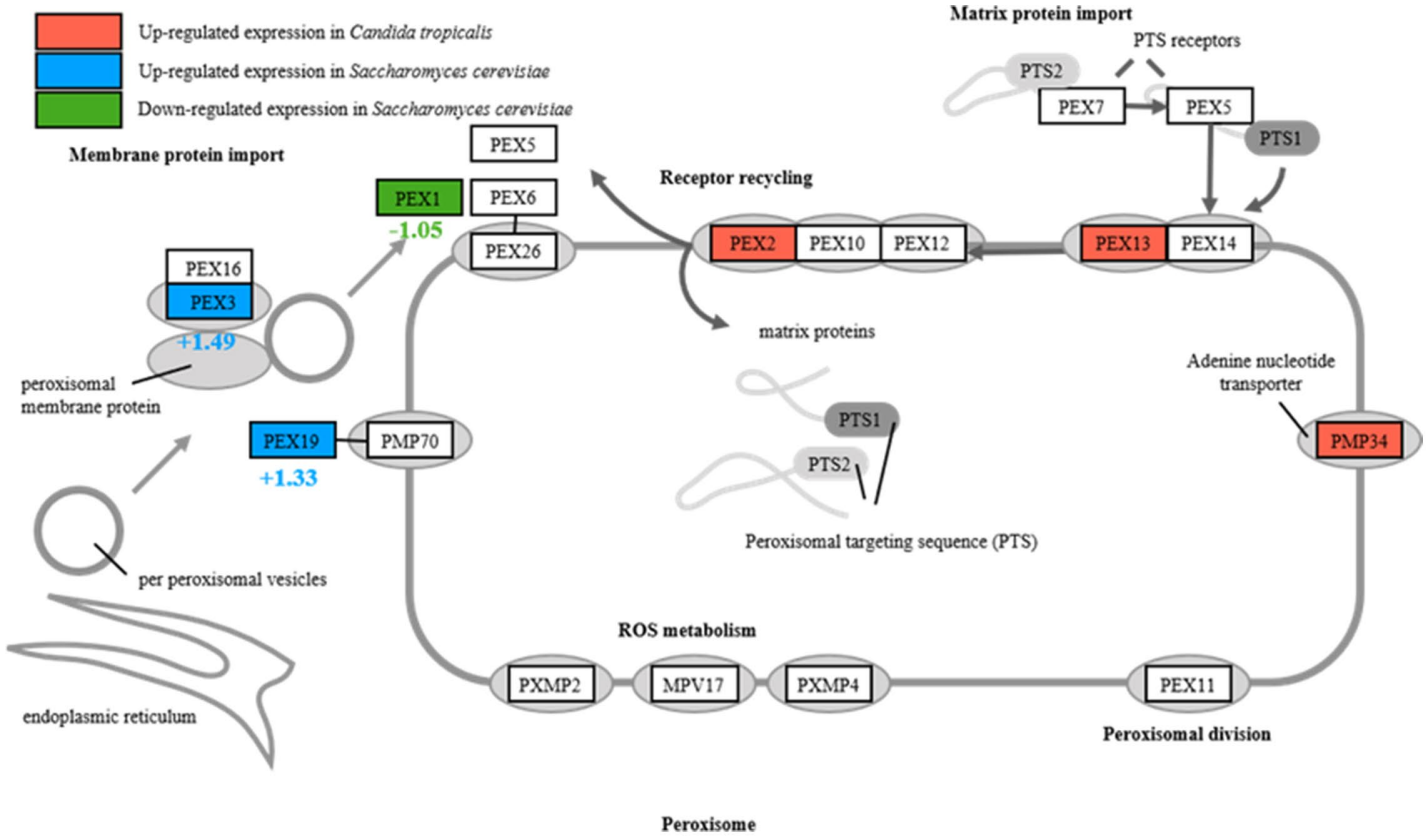
Differential expression of peroxisome in *Candida tropicalis* and *Saccharomyces cerevisiae.*

Further analysis of transcriptomic and metabolomic data to understand the distinct responses of the strains revealed significant upregulation of genes encoding Peroxisome, specifically *PEX2*, *PEX13*, and *PMP34*, in the peroxisome pathway of *C. tropicalis* SHC-03 (Table 1 and Figure 4). These proteins play crucial roles in the detoxification of reactive oxygen species, enhancing the cell’s ability to manage oxidative stress. In contrast, *S. cerevisiae* BY4742 displayed elevated gene expression levels exclusively for *PEX3* and *PEX19* within the same pathway, with fold changes of 1.49 and 1.33, respectively, (Figure 4). Notably, the expression of *PEX1* decreased by 1.05-fold compared to baseline (0 h), suggesting a more limited peroxisomal response under phenol stress (Figure 4). Additionally, *C. tropicalis* SHC-03 showed increased expression in pathways associated with the oxidative stress response, including Oxidoreductase activity, Iron ion binding, FAD binding, and Ascorbate and aldarate metabolism (Figure 2C), with metabolites in Ascorbate and aldarate also exhibited upregulation (Supplementary Figure S5). This suggests a more robust and nuanced response to phenol-induced stress in the strain SHC-03 compared to the strain BY4742, with the former displaying a superior ability to counteract oxidative stress from phenol.

**Table 1:** Transcription levels of differentially expressed genes on the peroxisome pathway in *Candida tropicalis* SHC-03.

Gene IDs	Product	EC number	Transcription levels
*CTRG_01657*	PEX2	-.-.-.-	1.50
*CTRG_05964*	PEX13	-.-.-.-	1.39
*CTRG_02480*	PMP34	-.-.-.-	1.25
*CTRG_04531*	PRDX5	1.11.1.24	1.14
*CTRG_02374*	ACOX	1.3.3.6	1.40
*CTRG_01068*	ACAA1	2.3.1.16	1.07
*CTRG_05829*	ACSL	6.2.1.3	2.14
*CTRG_01503*	ACSL	6.2.1.3	3.12
*CTRG_02265*	ACSL	6.2.1.3	1.43
*CTRG_05500*	ACSL	6.2.1.3	3.41
*CTRG_04642*	PDCR	1.3.1.124	1.02
*CTRG_05691*	ECH	5.3.3.21	1.80
*CTRG_03043*	PECI	5.3.3.8	1.96
*CTRG_03045*	PECI	5.3.3.8	1.38
*CTRG_00243*	CRAT	2.3.1.7	1.27
*CTRG_00457*	CRAT	2.3.1.7	2.12
*CTRG_04354*	CRAT	2.3.1.7	1.35
*CTRG_00909*	IDH	1.1.1.42	2.04

Transcription levels of the genes were represented by the values of log_2_ (fold change) at 6 h against the control (at 0 h).

The comparative analysis illustrated the disparate oxidative stress responses and phenol resilience between *C. tropicalis* SHC-03 and *S. cerevisiae* BY4742. Specifically, the strain SHC-03 showed a markedly enhanced capacity for mitigating oxidative stress triggered by phenol, underscoring the significant differences in stress adaptation mechanisms between the two yeast strains.

### 3.4 Metabolic adaptation to phenol stress in *C. tropicalis*

Despite the non-detection of catechol and cis, cis-muconate and minimal changes in the phenol concentration after 6 h, these findings do not negate the onset of phenol metabolism by *C. tropicalis*. The upregulation of phenylalanine metabolism and associated pathways suggests that the yeast is undergoing preliminary metabolic adjustments in preparation for phenol degradation (Figures 2C,D).

Unlike *S. cerevisiae*, which cannot metabolize phenol as a sole carbon source, *C. tropicalis* undergoes significant metabolic reprogramming under phenolic stress. Within 6 h of exposure to a phenol-rich medium, a notable suppression was observed in carbohydrate metabolism pathways such as glycolysis, gluconeogenesis, and the metabolism of fructose and mannose (Figure 2C and Supplementary Table S3). This suppression signifies a strategic shift from carbohydrate utilization, redirecting the yeast’s metabolic capacity to process phenol as an alternative carbon source.

Concurrently, there was a marked increase in the expression of genes related to membrane components and transmembrane transporters, which play a crucial role in enhancing the cell’s ability for substance exchange and signal transduction (Figure 2C). This upregulation improves the cellular interface for environmental interactions, crucial for adapting to phenolic stress. Specifically, genes encoding Major Facilitator Superfamily (MFS) transporters, including *CTRG_06081*, *CTRG_06100*, *CTRG_05562*, *CTRG_03960* and *CTRG_03958*, were significantly upregulated (Table 2). MFS transporters are integral membrane proteins that facilitate the passive transport of substrates such as sugars, polyols, drugs, and various organic acids across cellular membranes (dos Santos et al., 2014; Wang et al., 2018). In the context of phenol stress, these transporters likely play pivotal roles in modulating the cellular uptake of phenol and the efflux of its toxic metabolites, thereby enhancing cell survival and metabolic efficiency under stress conditions. Furthermore, our study observed a reduction in secondary metabolite synthesis, particularly in pathways for neomycin, kanamycin, and gentamicin biosynthesis (Figure 2D). The reduction suggests a strategic reallocation of cellular resources towards essential pathways for phenol detoxification and assimilation into the central metabolism.

**Table 2:** Transcription levels of differentially expressed genes on transmembrane transporter activity in *Candida tropicalis SHC-03.*

Gene IDs	Product	Transcription levels
*CTRG_00976*	MFS transporter	1.40
*CTRG_06081*	MFS transporter	6.29
*CTRG_05012*	MFS transporter	2.11
*CTRG_06100*	MFS transporter	4.76
*CTRG_06249*	MFS transporter	1.65
*CTRG_00968*	MFS transporter	1.86
*CTRG_05562*	MFS transporter	6.56
*CTRG_01806*	MFS transporter	2.06
*CTRG_04977*	MFS transporter	1.38
*CTRG_06212*	peptide transporter PTR2	5.43
*CTRG_01125*	choline transport protein	2.42
*CTRG_04615*	high-affinity glucose transporter	1.93
*CTRG_05890*	Uncharacterized MFS-type transporter	3.06
*CTRG_05874*	Uncharacterized MFS-type transporter	3.11
*CTRG_03730*	Uncharacterized MFS-type transporter	5.41
*CTRG_04909*	Uncharacterized MFS-type transporter	3.95
*CTRG_03958*	Uncharacterized MFS-type transporter	6.17
*CTRG_05344*	Uncharacterized MFS-type transporter	3.40
*CTRG_05953*	Uncharacterized MFS-type transporter	3.90
*CTRG_03960*	Uncharacterized MFS-type transporter	7.50
*CTRG_05923*	Uncharacterized MFS-type transporter	2.35
*CTRG_00662*	Uncharacterized MFS-type transporter	2.21
*CTRG_02833*	Uncharacterized MFS-type transporter	1.89
*CTRG_04557*	Uncharacterized MFS-type transporter	1.72
*CTRG_03602*	Uncharacterized MFS-type transporter	1.76
*CTRG_00558*	Uncharacterized MFS-type transporter	1.89
*CTRG_04303*	Uncharacterized MFS-type transporter	2.15
*CTRG_06168*	Uncharacterized MFS-type transporter	1.75

Transcription levels of the genes were represented by the values of log_2_ (fold change) at 6 h against the control (at 0 h).

**Figure 5:**
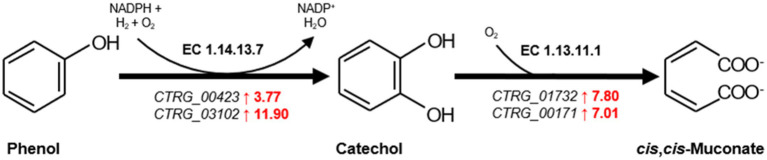
The first and second step of *β*-ketoadipate pathway in the biodegradation phenol. Genes significantly upregulated are highlighted in red. The numbers represent transcription levels, quantified as log2 (fold change) of gene expression at 6 h relative to the control at 0 h, indicating the magnitude of upregulation.

Furthermore, significant upregulation was noted in genes encoding Phenol 2-monoxygenase (*CTRG_00423* and *CTRG_03102*) were upregulated by 3.77-fold and 11.90-fold (Figure 5), initiating the degradation of phenol to catechol, a step that relies on NADPH. The increased expression of enzymes requiring NAD(P)H as a cofactor, especially those involved in oxidoreductive reactions (Supplementary Table S2), highlights a heightened demand for cofactors necessary for phenol metabolism. And the genes for Catechol 1,2-dioxygenase (*CTRG_01732* and *CTRG_00171*) were upregulated by 7.80-fold and 7.01-fold (Figure 5), respectively, indicating the activation of the *β*-ketoadipate pathway for catechol conversion to cis, cis-muconate (Figure 5) (Yan et al., 2005; Long et al., 2014; Wang et al., 2020; He et al., 2022).

## 4 Discussion

Our results demonstrating *C. tropicalis* SHC-03’s superior ability to degrade phenol, showing degradation rates of 99.47 and 95.91% at phenol concentrations of 1.6 g/L and 1.8 g/L respectively, highlight the presence of a robust enzymatic system in *C. tropicalis*. Meanwhile, Phenol 2-monoxygenase and Catechol 1,2-dioxygenase, are pivotal for initiating the breakdown of phenol into less toxic intermediates (Adav et al., 2007; Long et al., 2014; Wang et al., 2020; He et al., 2022). The observed growth inhibition in *C. tropicalis* SHC-03 at higher phenol concentrations of 2.0 g/L and 2.2 g/L, aligns with findings by He et al., who reported a threshold phenol concentration beyond which cellular activity is significantly hampered (He et al., 2022). This suggests that *C. tropicalis* SHC-03 possesses a threshold for phenol tolerance and degradation, beyond which the toxic effects of phenol outweigh the yeast’s enzymatic capacity for degradation. In contrast, the absence of phenol degradation in *S. cerevisiae* underlines a lack of such enzymatic pathways or an effective phenolic stress response. This aligns with observations that *S. cerevisiae* may require genetic modification to efficiently metabolize phenolic compounds (Adeboye, 2016), further underscoring the metabolic versatility and resilience of *C. tropicalis* SHC-03 in phenol degradation and stress response.

In this study, significant metabolic reprogramming of *C. tropicalis* was observed, characterized by the suppression of primary carbohydrate metabolism and the concurrent activation of phenol degradation pathways, indicates a sophisticated prioritization of metabolic resources under phenolic stress. This strategic shift, underscored by the suppression of glycolysis, gluconeogenesis, and fructose and mannose metabolism pathways, demonstrates the yeast’s capability to dynamically reroute its metabolic machinery towards the utilization of phenol as an alternative carbon source (He et al., 2022). Furthermore, the upregulation of Major Facilitator Superfamily (MFS) transporters, such as *CTRG_06081, CTRG_06100*, *CTRG_05562*, *CTRG_03960*, and *CTRG_03958*, underscores a vital cellular mechanism for managing phenolic compounds. MFS transporters are not merely passive conduits for molecular traffic but active participants in the cell’s response to environmental changes. They facilitate the uptake and secretion of a wide range of substrates, including sugars, ions, and various xenobiotics, thus playing a pivotal role in cellular adaptability to environmental stresses. The increased expression of these transporters in *C. tropicalis* SHC-03 not only facilitates more efficient phenol uptake and detoxification but also highlights a sophisticated regulatory network that enhances resilience and adaptability to environmental stresses (dos Santos et al., 2014; Wang et al., 2018). This finding is in line with Wang et al. (2018), who emphasized the crucial role of MFS transporters in microbial responses to xenobiotic stress, aiding in the efflux of toxic substances and thus safeguarding cellular integrity. The downregulation of secondary metabolite synthesis pathways, particularly those associated with antibiotic biosynthesis, suggests a strategic reallocation of cellular resources. This adaptation, aimed at ensuring survival under phenolic stress by diverting energy and molecular precursors towards phenol detoxification pathways, mirrors observations by Montibus et al. (2015). They demonstrated that environmental stressors prompt a significant redirection of metabolic fluxes, optimizing microorganisms’ survival strategies in adverse conditions. The collective insights from these studies reinforce the concept that *C. tropicalis*’ metabolic reprogramming in response to phenolic stress is not an isolated phenomenon but part of a broader, highly conserved strategy among microorganisms to cope with toxic environments.

*PEX2* functions as part of the RING complex, a ubiquitin ligase critical for the import of enzymes into peroxisomes, affecting the turnover and proliferation of these organelles and *PEX13* is a component of the docking complex required for the import of matrix proteins into peroxisomes, facilitating the efficient sequestration and breakdown of ROS, while *PEX34* is not a universally recognized PEX protein, assuming a similar role would suggest involvement in peroxisomal membrane assembly or function, potentially enhancing the compartmentalization and efficiency of peroxisomal processes (Jansen et al., 2021). The upregulation of these *PEX* genes in *C. tropicalis* suggests an enhanced capacity for peroxisome proliferation, import of antioxidative enzymes, and overall improved management of oxidative stress. This is particularly important under phenolic stress conditions, where elevated ROS levels can cause significant cellular damage. Conversely, *S. cerevisiae* exhibits upregulated expression of *PEX3* and *PEX19*, indicating active peroxisomal maintenance but possibly a less adaptable response to oxidative stresses. The specific patterns of *PEX* gene expression in *C. tropicalis* underscore its superior ability to manage oxidative stress, likely due to more dynamic peroxisomal adaptations. At the same time, the genes related to oxidoreductase activities, iron ion and FAD binding, ascorbic acid and uronate metabolism pathway in *C. tropicalis* were also up-regulated. And it underscored a comprehensive strategy not merely aimed at detoxifying ROS but also at maintaining cellular redox equilibrium and protecting cellular components from oxidative damage (Dias-Gunasekara et al., 2006; Herrero et al., 2008; Xiao et al., 2021). Enhanced oxidoreductase activities, pivotal in converting ROS into less reactive molecules, underscore a critical adaptive response to the oxidative stress resultant from phenol metabolism (Herrero et al., 2008). The roles of iron and FAD are particularly noteworthy, given their involvement in redox reactions and oxidative stress management, suggesting that *C. tropicalis* may optimize its redox balance as a countermeasure to phenolic stress (Dias-Gunasekara et al., 2006). Additionally, the upregulation of the ascorbate and aldarate metabolism pathways, accompanied by increased expression of related metabolites, signifies a strategic enhancement of antioxidant defenses, which further highlights *C. tropicalis*’s adaptive mechanisms to mitigate and thrive under phenolic-induced oxidative stress conditions (Xiao et al., 2021).

The upregulation of genes encoding Phenol 2-monoxygenase and Catechol 1,2-dioxygenase, pivotal for the initial steps in phenol catabolism, does not merely signify an enhanced capacity for phenol degradation. It also reflects an intricate regulatory adaptation that enables *C. tropicalis* to efficiently convert phenol into catechol and subsequently to cis, cis-muconate via the *β*-ketoadipate pathway, a mechanism that is central to the degradation of aromatic compounds (Yan et al., 2005; Long et al., 2014; Wang et al., 2020; He et al., 2022). The reliance on NAD(P)H in these reactions further illuminates the critical role of redox balance in the yeast’s response to phenolic stress, suggesting an integrated response that encompasses both the efficient degradation of phenol and the maintenance of cellular redox homeostasis (Long et al., 2014).

In summary, the metabolic adaptation of *C. tropicalis* to phenolic stress is emblematic of a highly evolved response that integrates phenol degradation with broader cellular objectives, including the maintenance of redox balance and cellular integrity. This orchestrated response not only underscores the yeast’s resilience to environmental stressors but also highlights its potential as a biotechnological tool for the bioremediation of phenolic pollutants.

## 5 Conclusion

This study illuminates the sophisticated metabolic and oxidative stress response mechanisms of *C. tropicalis* under phenolic stress. Unlike *S. cerevisiae*, which lacks the metabolic flexibility to degrade phenol, *C. tropicalis* demonstrates an exceptional ability to not only metabolize phenol efficiently but also to manage the resultant oxidative stress effectively. The research highlights the critical roles of Phenol 2-monoxygenase and Catechol 1,2-dioxygenase in phenol degradation, alongside a strategic metabolic shift that prioritizes the utilization of phenol as an alternative carbon source. Furthermore, the enhanced expression of *PEX* genes and pathways involved in the oxidative stress response underscores *C. tropicalis*’s comprehensive strategy for maintaining cellular homeostasis and integrity in the face of phenolic-induced stress. Our results contribute to the field of microbial bioremediation, providing valuable insights into the adaptive mechanisms that enable *C. tropicalis* to thrive in phenol-contaminated environments. In conclusion this research not only enhances our understanding of yeast biology and environmental adaptation but also underscores the potential of *C. tropicalis* as a biotechnological tool for the remediation of phenolic pollutants, offering a promising avenue for addressing environmental contamination challenges.

## Data Availability

The raw data supporting the findings of this study have been deposited in publicly accessible repositories. The transcriptome sequencing data have been uploaded to the NCBI Sequence Read Archive (SRA) under the accession number PRJNA1102611. The metabolomics data are available in the MetaboLights repository and can be accessed via the following link: https://www.ebi.ac.uk/metabolights with the accession number MTBLS10757.

## Footnotes

^1^ https://david.ncifcrf.gov/tools.jsp

^2^ https://www.metaboanalyst.ca/

^3^ https://pathways.embl.de/
