# Supplementary material for: Unveiling superior phenol detoxification and degradation ability in Candida tropicalis SHC-03: a comparative study with Saccharomyces cerevisiae BY4742
Source: Front Microbiol. 2024 Sep 11;15:1442235. doi: 10.3389/fmicb.2024.1442235 (PMC11441332; doi:10.3389/fmicb.2024.1442235)
Supplement: Supplementary file 1 [file Table_1.DOCX]

**Table S1 Statistical table of sequencing data**

| **Sample** | **Clean_reads** | **Clean_bases** | **Q20** | **Q30** | **GC_pct** |
| --- | --- | --- | --- | --- | --- |
| T1 | 45783042 | 6.87G | 97.92 | 93.81 | 36.59 |
| T2 | 46509000 | 6.98G | 97.74 | 923.34 | 36.78 |
| T3 | 44897970 | 6.73G | 97.89 | 93.77 | 36.89 |
| T4 | 44227304 | 6.63G | 97.78 | 93.65 | 36.61 |
| T5 | 42900248 | 6.44G | 97.43 | 92.86 | 36.97 |
| T6 | 43963020 | 6.59G | 97.68 | 93.39 | 36.88 |
| S1 | 39160766 | 5.87G | 97.21 | 92.6 | 41.02 |
| S2 | 37482488 | 5.62G | 96.98 | 92.16 | 40.88 |
| S3 | 41173892 | 6.18G | 97.23 | 92.54 | 40.92 |
| S4 | 39186138 | 5.88G | 97.11 | 92.39 | 41.57 |
| S5 | 38807290 | 5.82G | 97.14 | 92.5 | 41.63 |
| S6 | 39395236 | 5.91G | 97.15 | 92.56 | 41.66 |

Note: T1,T2,T3: 0 h of *Candida tropicalis* SHC-03 ; T4,T5,T6: 6 h of *Candida tropicalis;* S1,S2,S3: 0 h of *Saccharomyces cerevisiae* BY4742; S4,S5,S6: 6 h of *Saccharomyces cerevisiae* BY4742.


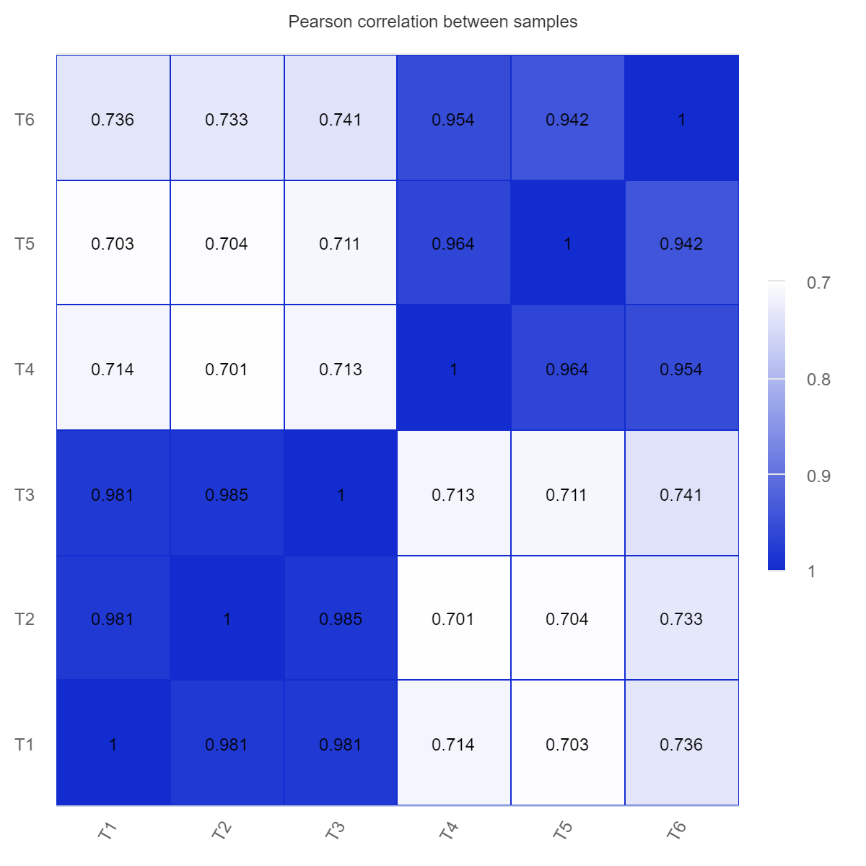


**Fig. S1.** Correlation analysis of *Candida tropicalis* SHC-03 samples

T1、T2、T3:0 h; T4、T5、T6:6 h.


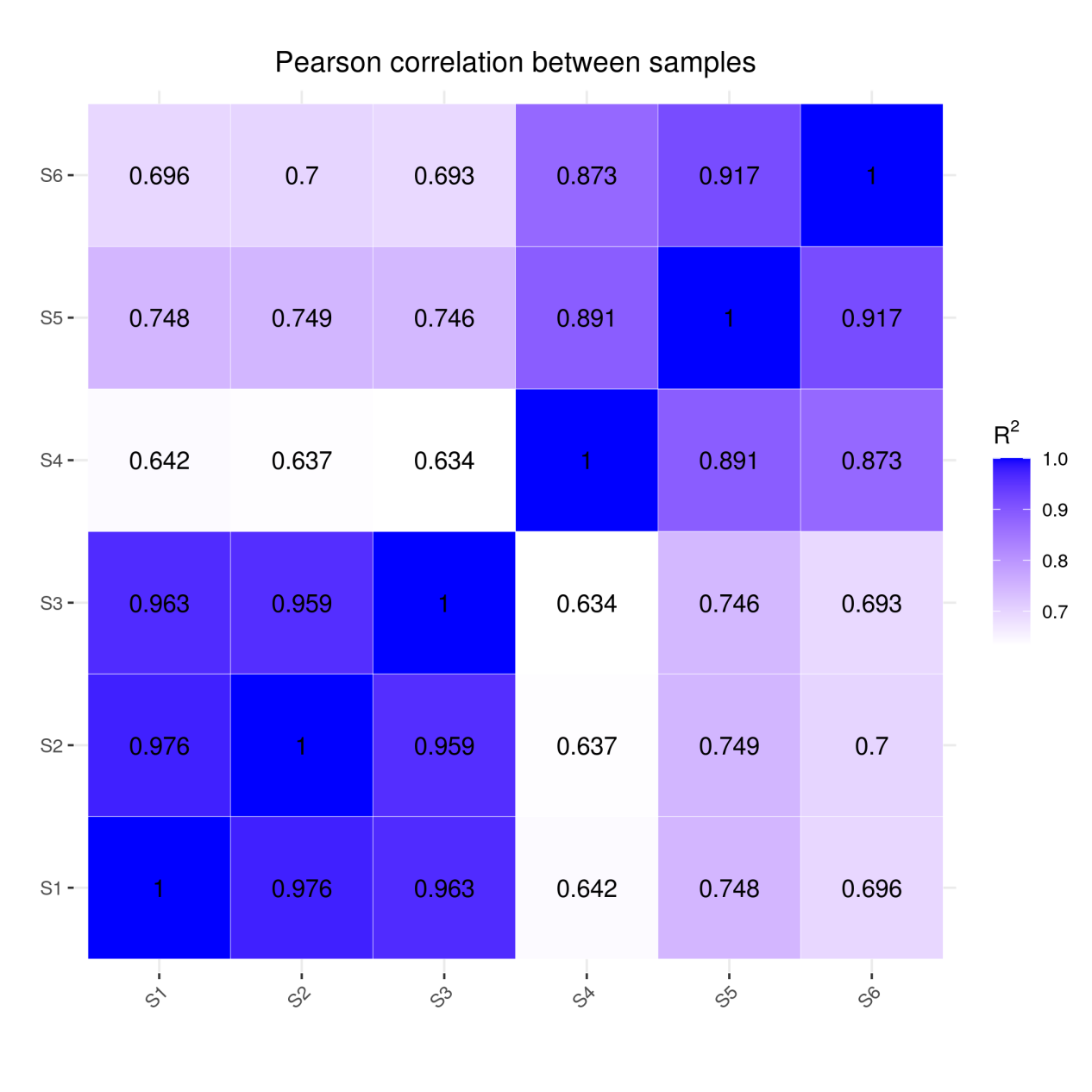


**Fig. S2.** Correlation analysis of *Saccharomyces cerevisiae* BY4742 samples

S1、S2、S3:0 h; S4、S5、S6:6 h.

**Table S2** **Enrichment table of upregulated genes in *Candids tropicalis* SHC-03**

| Term | pathway | Gene IDs |
| --- | --- | --- |
| GO:0016021 | integral component of membrane | *CTRG_00558, CTRG_02738, CTRG_01125, CTRG_01485, CTRG_03270, CTRG_03271, CTRG_05570, CTRG_05331, CTRG_05971, CTRG_03278, CTRG_02861, CTRG_02587, CTRG_03676, CTRG_00828, CTRG_04646, CTRG_04648, CTRG_04526, CTRG_01759, CTRG_00306, CTRG_03958, CTRG_02508, CTRG_03839, CTRG_00662, CTRG_01352, CTRG_05184, CTRG_06032, CTRG_02593, CTRG_05344, CTRG_03683, CTRG_03960, CTRG_03962, CTRG_02995, CTRG_03326, CTRG_03602, CTRG_04814, CTRG_00018, CTRG_00810, CTRG_01621, CTRG_01862, CTRG_01061, CTRG_01060, CTRG_02082, CTRG_02480, CTRG_06043, CTRG_06167, CTRG_06168, CTRG_05874, CTRG_05513, CTRG_04303, CTRG_03730, CTRG_06205, CTRG_05875, CTRG_04307, CTRG_02766, CTRG_00924, CTRG_00527, CTRG_00526, CTRG_00009, CTRG_03617, CTRG_04948, CTRG_04031, CTRG_05485, CTRG_06212, CTRG_03620, CTRG_06219, CTRG_04557, CTRG_03865, CTRG_01849, CTRG_05923, CTRG_00913, CTRG_02812, CTRG_04319, CTRG_00992, CTRG_00078, CTRG_00077, CTRG_00231, CTRG_00110, CTRG_05890, CTRG_03591, CTRG_05012, CTRG_03471, CTRG_06100, CTRG_05498, CTRG_05255, CTRG_05378, CTRG_03477, CTRG_03752, CTRG_04602, CTRG_03119, CTRG_02823, CTRG_02824, CTRG_02945, CTRG_00348, CTRG_01314, CTRG_01278, CTRG_01795, CTRG_01794, CTRG_01430, CTRG_00581, CTRG_06232, CTRG_03120, CTRG_03122, CTRG_02034, CTRG_03125, CTRG_03763, CTRG_05303, CTRG_03247, CTRG_04214, CTRG_03764, CTRG_04615, CTRG_02833, CTRG_04977, CTRG_03527, CTRG_02559, CTRG_01824, CTRG_02835, CTRG_05708, CTRG_00976, CTRG_00459, CTRG_00458, CTRG_00579, CTRG_05949, CTRG_05709, CTRG_00177, CTRG_01143, CTRG_06081, CTRG_00290, CTRG_02280, CTRG_04222, CTRG_04068, CTRG_02320, CTRG_04501, CTRG_03653, CTRG_05953, CTRG_06249, CTRG_03930, CTRG_04626, CTRG_02602, CTRG_00968, CTRG_02603, CTRG_02725, CTRG_00843, CTRG_00842, CTRG_01777, CTRG_01655, CTRG_04909, CTRG_00561, CTRG_01374, CTRG_00681, CTRG_00560, CTRG_00042, CTRG_04070, CTRG_05562, CTRG_04750, CTRG_04991, CTRG_04630, CTRG_03663, CTRG_05689, CTRG_04479, CTRG_05964, CTRG_02851, CTRG_05721, CTRG_03942, CTRG_01806, CTRG_02217, CTRG_00318, CTRG_02736, CTRG_05969* |
| GO:0005576 | extracellular region | *CTRG_00700, CTRG_03052, CTRG_00941, CTRG_03750, CTRG_03871, CTRG_01112, CTRG_02432, CTRG_05930, CTRG_03885, CTRG_02710, CTRG_02799, CTRG_01041* |
| GO:0009277 | fungal-type cell wall | *CTRG_06222, CTRG_06145, CTRG_02074, CTRG_05652, CTRG_00233, CTRG_02585, CTRG_04685, CTRG_02201, CTRG_02444, CTRG_01926, CTRG_05838, CTRG_01923* |
| GO:0005777 | peroxisome | *CTRG_01657, CTRG_02374, CTRG_00909, CTRG_06148, CTRG_01068, CTRG_00243, CTRG_05886, CTRG_02668, CTRG_01109* |
| GO:0009986 | cell surface | *CTRG_06222, CTRG_00233, CTRG_04685, CTRG_02201, CTRG_01926, CTRG_05838, CTRG_01923* |
| GO:0031225 | anchored component of membrane | *CTRG_06145, CTRG_00941, CTRG_03871, CTRG_03600, CTRG_03942, CTRG_01041* |
| GO:0022857 | transmembrane transporter activity | *CTRG_00976, CTRG_00558, CTRG_01125, CTRG_06081, CTRG_05890, CTRG_05012, CTRG_06100, CTRG_06168, CTRG_05874, CTRG_03278, CTRG_04303, CTRG_04501, CTRG_03730, CTRG_05953, CTRG_06249, CTRG_00968, CTRG_02603, CTRG_03958, CTRG_04909, CTRG_00662, CTRG_01374, CTRG_01352, CTRG_00042, CTRG_05562, CTRG_05344, CTRG_06212, CTRG_03960, CTRG_01806, CTRG_04557, CTRG_03602, CTRG_04615, CTRG_02833, CTRG_04977, CTRG_05923* |
| GO:0016491 | oxidoreductase activity | *CTRG_01556, CTRG_01730, CTRG_00882, CTRG_01595, CTRG_01034, CTRG_01110, CTRG_01363, CTRG_00580, CTRG_01152, CTRG_05482, CTRG_05388, CTRG_05423, CTRG_02102, CTRG_02532, CTRG_04478, CTRG_02995, CTRG_03611, CTRG_04712, CTRG_00508, CTRG_05989, CTRG_02834, CTRG_02736, CTRG_01824* |
| GO:0003824 | catalytic activity | *CTRG_03959, CTRG_01888, CTRG_01886, CTRG_00175, CTRG_01070, CTRG_05691, CTRG_03151, CTRG_06101, CTRG_06079, CTRG_04880, CTRG_03043, CTRG_03045, CTRG_05017, CTRG_02647* |
| GO:0005506 | iron ion binding | *CTRG_05032, CTRG_05906, CTRG_01129, CTRG_03120, CTRG_02663, CTRG_03864, CTRG_03930, CTRG_01061, CTRG_02725, CTRG_01060* |
| GO:0020037 | heme binding | *CTRG_03120, CTRG_02660, CTRG_05479, CTRG_03930, CTRG_05855, CTRG_01061, CTRG_02658, CTRG_02725, CTRG_01060* |
| GO:0000293 | ferric-chelate reductase activity | *CTRG_02738, CTRG_06043, CTRG_03839, CTRG_01655, CTRG_03122, CTRG_00110, CTRG_00681, CTRG_03527* |
| GO:0015171 | amino acid transmembrane transporter activity | *CTRG_00458, CTRG_03683, CTRG_04068, CTRG_05513, CTRG_05303, CTRG_03676, CTRG_04646, CTRG_02945* |
| GO:0004806 | triglyceride lipase activity | *CTRG_03052, CTRG_01278, CTRG_02861, CTRG_05930, CTRG_03885, CTRG_02799, CTRG_06185* |
| GO:0071949 | FAD binding | *CTRG_00423, CTRG_03917, CTRG_02660, CTRG_02374, CTRG_03102, CTRG_05855, CTRG_02658* |
| GO:0016620 | oxidoreductase activity, acting on the aldehyde or oxo group of donors, | *CTRG_04471, CTRG_05010, CTRG_00532, CTRG_01335, CTRG_01342* |
| GO:0005975 | carbohydrate metabolic process | *CTRG_03727, CTRG_03728, CTRG_00941, CTRG_01049, CTRG_01021, CTRG_06095, CTRG_01063, CTRG_00726, CTRG_02280* |
| GO:0016042 | lipid catabolic process | *CTRG_03052, CTRG_01278, CTRG_05930, CTRG_03885, CTRG_02799, CTRG_06185* |
| GO:0006826 | iron ion transport | *CTRG_02738, CTRG_06043, CTRG_03122, CTRG_00110, CTRG_03527* |
| GO:0016226 | iron-sulfur cluster assembly | *CTRG_00833, CTRG_05032, CTRG_01129, CTRG_02663, CTRG_02766* |
| ctp01100 | Metabolic pathways | *CTRG_01888, CTRG_01007, CTRG_01886, CTRG_00797, CTRG_01762, CTRG_01484, CTRG_02180, CTRG_05570, CTRG_04880, CTRG_02861, CTRG_02102, CTRG_04402, CTRG_02104, CTRG_02501, CTRG_01595, CTRG_01110, CTRG_03162, CTRG_05500, CTRG_03326, CTRG_05748, CTRG_02998, CTRG_01109, CTRG_03726, CTRG_05906, CTRG_03727, CTRG_01503, CTRG_00898, CTRG_03728, CTRG_00532, CTRG_01861, CTRG_01068, CTRG_01342, CTRG_00890, CTRG_01063, CTRG_01062, CTRG_02361, CTRG_03178, CTRG_05479, CTRG_03338, CTRG_02888, CTRG_04702, CTRG_00924, CTRG_00009, CTRG_01733, CTRG_02927, CTRG_01335, CTRG_00882, CTRG_05241, CTRG_05482, CTRG_02374, CTRG_02770, CTRG_04035, CTRG_04279, CTRG_03628, CTRG_01049, CTRG_00232, CTRG_06185, CTRG_05010, CTRG_06101, CTRG_03474, CTRG_00909, CTRG_02265, CTRG_05017, CTRG_00902, CTRG_02668, CTRG_04729, CTRG_03519, CTRG_01793, CTRG_06079, CTRG_02032, CTRG_05388, CTRG_05423, CTRG_02795, CTRG_03247, CTRG_02436, CTRG_04617, CTRG_05708, CTRG_05829, CTRG_05949, CTRG_03927, CTRG_02838, CTRG_00610, CTRG_01385, CTRG_01021, CTRG_00294, CTRG_00171, CTRG_03135, CTRG_03653, CTRG_04984, CTRG_03930, CTRG_04986, CTRG_04626, CTRG_00726, CTRG_04627, CTRG_05958, CTRG_01779, CTRG_03939, CTRG_00680, CTRG_01371, CTRG_04471, CTRG_05320, CTRG_04750, CTRG_02333, CTRG_03389, CTRG_05721, CTRG_02736* |
| ctp01110 | Biosynthesis of secondary metabolites | *CTRG_05708, CTRG_03628, CTRG_01007, CTRG_00532, CTRG_01861, CTRG_01068, CTRG_00232, CTRG_01342, CTRG_01385, CTRG_01484, CTRG_01021, CTRG_01062, CTRG_06185, CTRG_05010, CTRG_05570, CTRG_02361, CTRG_03178, CTRG_00909, CTRG_03653, CTRG_02104, CTRG_03338, CTRG_00726, CTRG_02888, CTRG_02668, CTRG_04702, CTRG_00924, CTRG_05958, CTRG_04729, CTRG_03519, CTRG_01335, CTRG_00882, CTRG_01595, CTRG_01793, CTRG_00580, CTRG_04471, CTRG_05482, CTRG_04750, CTRG_02032, CTRG_02374, CTRG_02795, CTRG_03389, CTRG_04478, CTRG_01109, CTRG_03407, CTRG_03726* |
| ctp00620 | Pyruvate metabolism | *CTRG_04729, CTRG_01888, CTRG_01007, CTRG_01886, CTRG_00532, CTRG_01335, CTRG_00882, CTRG_01595, CTRG_01342, CTRG_01021, CTRG_01062, CTRG_04471, CTRG_05010, CTRG_05482, CTRG_05320, CTRG_04880, CTRG_02032, CTRG_03389, CTRG_05017, CTRG_02436* |
| ctp04146 | Peroxisome | *CTRG_05829, CTRG_01657, CTRG_01503, CTRG_00457, CTRG_01068, CTRG_00243, CTRG_02480, CTRG_05691, CTRG_03043, CTRG_04354, CTRG_03045, CTRG_02374, CTRG_00909, CTRG_05500, CTRG_04642, CTRG_02265, CTRG_04531, CTRG_05964, CTRG_02756* |
| ctp01200 | Carbon metabolism | *CTRG_03628, CTRG_04729, CTRG_02838, CTRG_01484, CTRG_01021, CTRG_01062, CTRG_05570, CTRG_02032, CTRG_05388, CTRG_02374, CTRG_03178, CTRG_00909, CTRG_02795, CTRG_02333, CTRG_03389, CTRG_00726, CTRG_02668, CTRG_04702, CTRG_03726* |
| ctp00071 | Fatty acid degradation | *CTRG_05829, CTRG_01503, CTRG_00532, CTRG_01335, CTRG_00882, CTRG_01068, CTRG_01595, CTRG_01342, CTRG_04471, CTRG_05010, CTRG_05482, CTRG_03043, CTRG_03045, CTRG_02374, CTRG_05500, CTRG_02265, CTRG_05958* |
| ctp00010 | Glycolysis / Gluconeogenesis | *CTRG_04729, CTRG_00532, CTRG_01335, CTRG_00882, CTRG_00232, CTRG_01595, CTRG_01342, CTRG_01062, CTRG_04471, CTRG_05010, CTRG_05482, CTRG_05570, CTRG_02361, CTRG_02032, CTRG_03726* |
| ctp00330 | Arginine and proline metabolism | *CTRG_04471, CTRG_05010, CTRG_03939, CTRG_02927, CTRG_00532, CTRG_01335, CTRG_01762, CTRG_01342, CTRG_01484, CTRG_04984, CTRG_04986, CTRG_02668* |
| ctp01212 | Fatty acid metabolism | *CTRG_05241, CTRG_05829, CTRG_01503, CTRG_01007, CTRG_02374, CTRG_03474, CTRG_05500, CTRG_02265, CTRG_01068, CTRG_05479, CTRG_02501, CTRG_05958* |
| ctp00360 | Phenylalanine metabolism | *CTRG_01779, CTRG_03939, CTRG_01793, CTRG_01484, CTRG_04984, CTRG_00680, CTRG_03930, CTRG_04986, CTRG_02668* |

**Table S3 Enrichment table of downregulated genes in *Candids tropicalis* SHC-03**

| ID | Term | Associated Genes Found |
| --- | --- | --- |
| GO:0005739 | mitochondrion | *CTRG_02937, CTRG_03509, CTRG_03709, CTRG_01368, CTRG_00376, CTRG_00211, CTRG_05690, CTRG_02083, CTRG_04464, CTRG_02682, CTRG_05115, CTRG_03454, CTRG_02069, CTRG_05955, CTRG_04326, CTRG_03911, CTRG_03978, CTRG_00789, CTRG_00601, CTRG_04807, CTRG_00564, CTRG_00067, CTRG_00760, CTRG_06094, CTRG_04492, CTRG_03582, CTRG_03285, CTRG_03001, CTRG_04993, CTRG_05367, CTRG_02117* |
| GO:0005886 | plasma membrane | *CTRG_00501, CTRG_00886, CTRG_00356, CTRG_00004, CTRG_01454, CTRG_01186, CTRG_01372, CTRG_02293, CTRG_04296, CTRG_06015, CTRG_05740, CTRG_02164, CTRG_03002, CTRG_02298, CTRG_05951, CTRG_01909, CTRG_05920, CTRG_05611, CTRG_05416, CTRG_05549* |
| GO:0005840 | ribosome | *CTRG_03509, CTRG_01025, CTRG_03070, CTRG_04492, CTRG_04243, CTRG_05731, CTRG_03454, CTRG_04885, CTRG_04338, CTRG_02534, CTRG_05657, CTRG_02667, CTRG_01738* |
| GO:0009277 | fungal-type cell wall | *CTRG_01856, CTRG_01855, CTRG_00149, CTRG_02140, CTRG_03262, CTRG_00169, CTRG_02154, CTRG_05456, CTRG_00155, CTRG_00154, CTRG_05458, CTRG_04899* |
| GO:0030479 | actin cortical patch | *CTRG_00965, CTRG_05373, CTRG_05168, CTRG_02164, CTRG_00630, CTRG_05955, CTRG_01186, CTRG_01716, CTRG_00746* |
| GO:0000786 | nucleosome | *CTRG_00676, CTRG_00675, CTRG_04732, CTRG_02624, CTRG_02625* |
| GO:0006412 | translation | *CTRG_01546, CTRG_01489, CTRG_01025, CTRG_01024, CTRG_00470, CTRG_03070, CTRG_04231, CTRG_05561, CTRG_04243, CTRG_02731, CTRG_02534, CTRG_01707, CTRG_00948, CTRG_05657, CTRG_02667, CTRG_01738, CTRG_03506* |
| GO:0006096 | tglycolytic process | *CTRG_00414, CTRG_04184, CTRG_00601, CTRG_03163, CTRG_02937, CTRG_00211, CTRG_05666, CTRG_01175* |
| GO:0006094 | gluconeogenesis | *CTRG_00601, CTRG_05608, CTRG_02937, CTRG_00259, CTRG_00211, CTRG_01175* |
| GO:0032543 | mitochondrial translation | *CTRG_00789, CTRG_06189, CTRG_04807, CTRG_00760, CTRG_03454, CTRG_04993, CTRG_03553* |
| GO:0000079 | regulation of cyclin-dependent protein serine/threonine kinase activity | *CTRG_05363, CTRG_05292, CTRG_02702, CTRG_03170, CTRG_04380* |
| GO:0035268 | protein mannosylation | *CTRG_01215, CTRG_04494, CTRG_05997, CTRG_03324, CTRG_05996* |
| GO:0046354 | mannan biosynthetic process | *CTRG_01215, CTRG_04494, CTRG_05997, CTRG_03324, CTRG_05996* |
| GO:0000030 | mannosyltransferase activity | *CTRG_00566, CTRG_05630, CTRG_05631, CTRG_01211, CTRG_05406, CTRG_05629, CTRG_01604* |
| GO:0003735 | structural constituent of ribosome | *CTRG_01546, CTRG_01025, CTRG_01024, CTRG_00470, CTRG_03070, CTRG_05192, CTRG_06189, CTRG_04231, CTRG_05352, CTRG_05561, CTRG_04243, CTRG_03454, CTRG_03553, CTRG_02731, CTRG_02534, CTRG_01707, CTRG_00948, CTRG_05657, CTRG_02667, CTRG_01738, CTRG_03506* |
| GO:0046961 | proton-transporting ATPase activity, rotational mechanism | *CTRG_00643, CTRG_05115, CTRG_03894, CTRG_02148, CTRG_06094* |
| GO:0051015 | actin filament binding | *CTRG_00965, CTRG_00630, CTRG_05955, CTRG_01716, CTRG_05749* |
| GO:0030170 | pyridoxal phosphate binding | *CTRG_03472, CTRG_00016, CTRG_01368, CTRG_04892, CTRG_00013, CTRG_02233, CTRG_05950, CTRG_05854, CTRG_02202, CTRG_03478, CTRG_01571* |
| GO:0004553 | hydrolase activity, hydrolyzing O-glycosyl compounds | *CTRG_02140, CTRG_03297, CTRG_00169, CTRG_04664, CTRG_04334, CTRG_02279, CTRG_02108* |
| ctp01100 | Metabolic pathways | *CTRG_03826, CTRG_05608, CTRG_01368, CTRG_01761, CTRG_01086, CTRG_03273, CTRG_05456, CTRG_05854, CTRG_04644, CTRG_03951, CTRG_00945, CTRG_02108, CTRG_05738, CTRG_02748, CTRG_00789, CTRG_00547, CTRG_04807, CTRG_03163, CTRG_04892, CTRG_02112, CTRG_02233, CTRG_03324, CTRG_01907, CTRG_00414, CTRG_00259, CTRG_00016, CTRG_00013, CTRG_00254, CTRG_01340, CTRG_01460, CTRG_01185, CTRG_04781, CTRG_02120, CTRG_03297, CTRG_02881, CTRG_05115, CTRG_04664, CTRG_05997, CTRG_05996, CTRG_02525, CTRG_05879, CTRG_00649, CTRG_05917, CTRG_01215, CTRG_00643, CTRG_01575, CTRG_00484, CTRG_01571, CTRG_01175, CTRG_04790, CTRG_04033, CTRG_06177, CTRG_03188, CTRG_02650, CTRG_04677, CTRG_05888, CTRG_04317, CTRG_00517, CTRG_02937, CTRG_00996, CTRG_03908, CTRG_00597, CTRG_04440, CTRG_03472, CTRG_03594, CTRG_06226, CTRG_02024, CTRG_03873, CTRG_03478, CTRG_02148, CTRG_03910, CTRG_03911, CTRG_04291, CTRG_00061, CTRG_06072, CTRG_05262, CTRG_02031, CTRG_04692, CTRG_06113, CTRG_06234, CTRG_05783, CTRG_03089, CTRG_04334, CTRG_02310, CTRG_05666, CTRG_00979, CTRG_01309, CTRG_05828, CTRG_00336, CTRG_00211, CTRG_01020, CTRG_06083, CTRG_06088, CTRG_04184, CTRG_04462, CTRG_06122, CTRG_03131, CTRG_02560, CTRG_02682, CTRG_03894, CTRG_02202, CTRG_02048, CTRG_02324, CTRG_04865, CTRG_04502, CTRG_01816, CTRG_00601, CTRG_00841, CTRG_00169, CTRG_01499, CTRG_00564, CTRG_06094, CTRG_05282, CTRG_03661, CTRG_06138, CTRG_02332* |
| ctp01110 | Biosynthesis of secondary metabolites | *CTRG_03826, CTRG_05608, CTRG_02937, CTRG_00597, CTRG_03472, CTRG_03594, CTRG_02024, CTRG_03951, CTRG_03478, CTRG_03911, CTRG_02108, CTRG_00945, CTRG_05738, CTRG_00789, CTRG_04291, CTRG_00061, CTRG_06072, CTRG_03163, CTRG_02031, CTRG_04892, CTRG_06113, CTRG_06234, CTRG_02233, CTRG_05666, CTRG_00414, CTRG_05828, CTRG_00016, CTRG_00211, CTRG_00013, CTRG_01020, CTRG_01460, CTRG_06083, CTRG_04184, CTRG_06122, CTRG_03297, CTRG_02881, CTRG_05115, CTRG_02202, CTRG_02048, CTRG_03777, CTRG_00649, CTRG_00569, CTRG_00601, CTRG_00841, CTRG_01575, CTRG_01175, CTRG_06094, CTRG_05282, CTRG_04790, CTRG_03661, CTRG_06138, CTRG_02650, CTRG_02898, CTRG_00517* |
| ctp01230 | Biosynthesis of amino acids | *CTRG_05608, CTRG_02937, CTRG_00259, CTRG_00016, CTRG_01575, CTRG_00211, CTRG_01175, CTRG_01460, CTRG_04291, CTRG_00061, CTRG_06094, CTRG_05282, CTRG_04184, CTRG_03163, CTRG_06234, CTRG_06138, CTRG_02650, CTRG_05115, CTRG_02233, CTRG_05666, CTRG_03911, CTRG_00945* |
| ctp03010 | Ribosome | *CTRG_01546, CTRG_01025, CTRG_01024, CTRG_00364, CTRG_05192, CTRG_04231, CTRG_05561, CTRG_04243, CTRG_05731, CTRG_04885, CTRG_02731, CTRG_02534, CTRG_01707, CTRG_00948, CTRG_05657, CTRG_02667, CTRG_01738, CTRG_03506, CTRG_02516ctp01200* |
| ctp01200 | Carbon metabolism | *CTRG_00547, CTRG_00414, CTRG_00601, CTRG_05608, CTRG_02937, CTRG_00259, CTRG_00016, CTRG_00211, CTRG_01175, CTRG_01460, CTRG_06094, CTRG_04184, CTRG_03163, CTRG_05115, CTRG_02233, CTRG_02024, CTRG_05666, CTRG_05888, CTRG_05738* |
| ctp00500 | Starch and sucrose metabolism | *CTRG_00414, CTRG_00601, CTRG_00996, CTRG_00169, CTRG_01020, CTRG_06083, CTRG_04790, CTRG_03297, CTRG_04892, CTRG_04334, CTRG_04664, CTRG_02048, CTRG_03951, CTRG_00517, CTRG_00649, CTRG_02108* |
| ctp00010 | Glycolysis / Gluconeogenesis | *CTRG_03826, CTRG_00414, CTRG_00601, CTRG_05608, CTRG_02937, CTRG_00211, CTRG_01175, CTRG_01460, CTRG_06094, CTRG_04184, CTRG_03163, CTRG_06113, CTRG_05115, CTRG_05666, CTRG_05738* |
| ctp00680 | Methane metabolism | *CTRG_00547, CTRG_04184, CTRG_03163, CTRG_05115, CTRG_00211, CTRG_01175, CTRG_05888, CTRG_06094* |
| ctp00051 | Fructose and mannose metabolism | *CTRG_00414, CTRG_05608, CTRG_03131, CTRG_03594, CTRG_05115, CTRG_00211, CTRG_06094* |
| ctp00650 | Butanoate metabolism | *CTRG_00569, CTRG_03472, CTRG_02332, CTRG_02202, CTRG_03478, CTRG_01571* |

**Table S4 Enrichment table of upregulated genes in *Saccharomyces cerevisiae* BY4742**

| Term | Pathway | Genes |
| --- | --- | --- |
| GO:0042026 | protein refolding | *MGE1, HSP82, HSP60, HSP104, SSC1, APJ1, SSE1, SSE2, CPR6, HSC82, SSA3, SSA4, KAR2, CNS1, SSA1, SSA2, HSP10, MDJ1, XDJ1, YDJ1, HSP78* |
| GO:0031118 | rRNA pseudouridine synthesis | *SNR191, SNR11, SNR44, SNR85, SNR86, SNR31, SNR9, SNR84, SNR8, SNR82, CIC1, SNR37, NHP2, SNR5, NOP10* |
| GO:0006457 | protein folding | *MGE1, HSP82, HSP60, PHB1, HCH1, APJ1, FPR1, SSC1, YME1, SSE1, SSE2, CUR1, CPR6, HSC82, STI1, SSA3, SSA4, SSA1, SSA2, HSP10, MDJ1, YDJ1, HSP12, CCT5, BUD27, AHA1, BTN2, SIS1, ZIM17, HSP26, FLC2, CNS1, XDJ1, HSP42* |
| GO:0051085 | chaperone mediated protein folding requiring cofactor | *HSP82, HSP104, AHA1, CUR1, HSC82, SIS1, HSP26, UBC4, NMA111, HSP12, HSP78, DDR2, HSP30* |
| GO:0034605 | cellular response to heat | *HSP82, HSP104, AHA1, CUR1, HSC82, SIS1, HSP26, UBC4, NMA111, HSP12, HSP78, DDR2, HSP30* |
| GO:0042254 | ribosome biogenesis | *RRP7, PXR1, ALB1, RPC19, BUD20, RPA12, KRI1, NPL3, RPA14, RRP9, RPS9A, MTG2, RRP14, RRP12, CGR1, RIO1, UTP15, IMP4, CIC1, NHP2, NAF1, ARB1, LCP5, NOP4, NOP1, NIP7, SNU13, RPB10, RPA34, RPF2, SNR37, EMG1, IPI3, BMS1, RRS1, MRT4, UTP5, NOP15, NOP16, PNO1, NOG2, DIM1, SPB4, TMA23, SPB1, ENP1, RCL1, ESF1, DBP8, NOP53, NOP10, DBP3, DBP2* |
| GO:0032543 | mitochondrial translation | *MRPS17, MRPS18, MRPS16, IFM1, SWS2, 15S_RRNA, RSM22, MRPL19, MSK1, MRP10, IMG1, IMG2, MRPL13, MRPL33, 21S_RRNA, MRP17, MRPL8, MRX14, YML6, RSM28, MRPL9, RSM27, MHR1, MEF2, RSM10, MNP1, MRPL27, MRPL28, MRP20, MRPL24, MRPL44, MRPL22, MRPL31, RSM18, GTF1, RSM19* |
| GO:0009060 | aerobic respiration | *MAM33, JAC1, QCR10, AI2, COQ5, QCR7, QCR6, PUF3, AI5_ALPHA, COX11, COX1, COX13, QCR9, MIX17, QCR8, CBP1, PAH1* |
| GO:0006458 | de novo' protein folding | *HSP82, HSP60, MDJ1, YDJ1, HSC82* |
| GO:0043335 | protein unfolding | *HSP104, SSC1, APJ1, HSP78, SPF1, ARB1* |
| GO:0005634 | nucleus | *POP6, RRP7, POP8, MCA1, YBL036C, NUP100, HAL9, LCD1, MFG1, RRN11, UBP13, UBP12, TOD6, YGR126W, SMC3, DOT6, UPC2, RRP9, YOR338W, PZF1, LSM12, YER084W, ARG80, SET2, WIP1, STP4, STP3, RTS3, STP1, MUD1, UFO1, YGR201C, HBN1, YBR085C-A, ARB1, CUB1, PHD1, RPN4, ASF1, FPR1, SNU13, FKH2, REC107, IPI3, FCY1, RRS1, VHS1, YDR034C-D, RAD28, ATG5, URH1, APA1, ASG1, SOH1, SSP2, PNO1, STR3, SUT2, CDC8, ENP1, CDC7, SNF6, SNU114, BDF2, RAD10, SDS3, IES2, VHR1, RAD14, IES5, IES6, UBI4, SOK1, URK1, SOK2, RAS2, HST1, KRI1, CDD1, IME1, YJR056C, SPC105, RRP14, RRP12, APA2, CSA1, MET22, SLD2, HSP104, PLM2, RMI1, MCD1, YDR261C-D, YNL108C, GPP1, APC11, HPM1, SLD3, DED1, BDH2, KIN28, CKS1, CTF3, GZF3, MAC1, URM1, NIP7, MRX14, CIP1, MEX67, YPL071C, MHR1, CSE4, CSE2, UTP5, ASK1, SOL1, DIM1, YKR018C, GCY1, SPO1, TIP41, NGL1, UNG1, CTH1, YHR127W, HEM12, NAB2, TBS1, UBP9, UBP7, CDH1, UBP3, RLM1, NMA111, PRP46, CTI6, YNL035C, MBF1, GRX1, URN1, YER079W, CYS3, GAL80, FLO8, NSE4, YDR365W-B, SAS5, NHP2, XDJ1, MIX17, MET30, YMR027W, SPT15, SDS23, MGT1, CBF2, VTS1, ORC2, BCK2, YDJ1, COA4, NOP15, NOP16, YMR114C, CTK1, BTN2, GLN1, KSP1, GLN3, YSF3, DAL81, DAL82, MRH4, PIN3, ZIP2, DAL80, BFR2, NOP10, BNA6, ALB1, YLR287C, FHL1, INO4, APJ1, INO2, YML082W, YAF9, SWC4, MER1, EXO5, HSP12, DPB2, ADR1, CGR1, SMP1, RFC3, PDE2, SPT8, IML2, NRG1, GFD1, SNU23, LOG1, HSP26, RAD34, KCS1, MOD5, NAF1, NDL1, LCP5, TMA10, LRP1, MOG1, CUP2, NUP1, YJR146W, ASR1, YER034W, NFS1, KAR2, SWE1, SPP381, CCL1, MET4, TRM112, SNT1, HUB1, SUA7, YDR514C, YNL134C, CSM4, CSM3, TMA23, CSM2, LRO1, MND2, BRN1, MSN1, OXR1, MSN2, REC8, TYE7, RAD51, YPL068C, ESF1, NOP53, ROX3, ACS1, PXR1, RPC17, CSN9, RPC19, TFB1, HED1, UBX6, CUR1, CDC21, PHO81, HTB2, RIO1, SKP1, MTR2, CHZ1, HMS2, MDM35, SKN7, IMP4, CDC40, NCE103, CDC31, YOR283W, CDC34, YOR192C-B, SUB1, NBL1, RPC37, HSP82, CLB5, RPB10, JJJ3, CTR1, SSA3, SSA4, SSA1, SSA2, THI4, RHO5, GRC3, PBI2, RGM1, TAH11, KTI11, NOG2, EAF3, KAP95, AHA1, EAF6, BOL2, NRM1, EAF7, YRM1, RSC4, STB1, RSC3, SDC1, SFG1, NSR1, YBR090C, ECM2, GIS3, YNG2, NPL3, CSR2, CRP1, PFS1, FYV6, SMX3, NEW1, FOB1, UFD1, ITC1, PAI3, ULP1, NQM1, YOR131C, YRF1-3, GDH3, RTG1, CMG1, DUS1, TDA9, SKS1, POC4, PCL8, SLU7, DCP1, PAH1, HIR1, KNS1, RAD9, PTP2, BAT2, YAP5, RAD6, YAP6, SSE1, SSE2, SRC1, SHP1, SLX5, RTT105, RTT107, RTT102, ELA1, HSF1, SFL1, MRT4, IFH1, UFD4, COM2, PDP3, DBP8, DBP3, YDL124W, DBP2, HCH1, CWC23, CWC24, GCD14, DST1, MGA1, YAR1, SIT4, RGS2, YPL257W-B, RPS9A, CWC21, GNA1, HAC1, ALG14, CIC1, SEN34, ULS1, PAL2, SIS1, SHQ1, RRG9, GPD2, NAR1, HIT1, PRP2, IDI1, NOP4, IGO1, IFM1, IGO2, NOP1, RPF2, HTL1, EMG1, RPN14, RPN13, YJR096W, LGE1, SPB4, SPB1, LEA1, RCL1, THP2, SKY1, YHR054C, NAT4, SPO14, BUD20, UBC6, RPA12, LDB7, CAF120, RPA14, MSS116, SNT309, BUD23, UTP15, MIH1, MOT3, BUD31, PKC1, IXR1, FPK1, HSK3, PCT1, BUD32, UBC4, NPT1, TAD2, SHB17, LIN1, YLR227W-B, LCB5, RPA34, RRN6, RRN7, DIF1, RDS3, IOC4, LSB1, BMS1, TGS1, RPC82, ALT2, IRC25, BUB3, GLC8, TIS11, BUB1, JEM1, ATC1, SSL2, ROG1, GSP1, ADE3, YIG1, CNS1, FUN30, ZPR1, HSH49* |
| GO:0012505 | endomembrane system | *DAP1, SPO14, SSO1, SRC1, SNC2, SEN34, YPT53, YPT52, YPT10, VPS21, VPS24, RHO5, PEP12, YPT1* |
| GO:0005739 | mitochondrion | *HSP60, PHB1, MIM1, MIM2, HAL9, YEA6, QCR10, KHA1, CDC21, SUC2, SHE10, IDP3, RIO1, SRL4, ATG3, STP4, COG7, MSP1, IMP2, CMC4, MDM35, GTT1, FAA1, NCE103, TOM22, VPS21, JEN1, UPS3, LIP2, UPS1, RCI37, ADY2, MPM1, MRPL19, FPR1, YMC2, MRPL13, YIL055C, ADH3, COX7, SSA2, FAU1, SUV3, TIM13, COX1, ENA2, ATG8, RHO5, HSP78, TIM12, TIM17, RHO1, CTA1, MEF2, EHT1, AAT1, GIP3, MRPL27, MRPL28, COI1, MRPL24, MRPL22, MRPL31, GTF1, FAT3, TIM22, TIM23, NSR1, MRPS17, MRS2, MRPS18, MRPS16, RAS2, SSC1, YME1, OPI3, MRPL33, ACH1, NEW1, MTG2, SPC105, GPA2, MIR1, CBP1, VRG4, TDA5, YKL070W, NCA3, RPO41, MCD1, YRF1-3, GDH3, MRP20, GPI18, LYS4, MSS51, MRPL44, MRX21, ZIM17, OMS1, DPI8, BAT2, ATP23, DJP1, ODC1, MRP10, BI2, BI4, IMG1, IMG2, MRX10, HYR1, MRP17, MRX14, INA22, ICT1, DLD3, MIC12, HSF1, ATP17, IRA2, SDH6, MIC10, MDJ1, ATP15, MHR1, ATP18, CRC1, PET100, TIM9, ATP11, UFD4, STF1, INH1, MIC27, NGL1, TEF2, DBP2, MAM33, MCO10, ISU1, UNG1, PUT1, SAT4, AI1, AI2, TBS1, PMD1, UBP3, AEP3, AEP1, MIX23, MBF1, HEM25, ATO2, CHA1, SEN34, YNK1, PDR5, ULS1, RRG9, GPD2, RRG8, FMP52, XDJ1, CIS1, MIX17, COX19, IFM1, COX15, MMO1, COX17, MSK1, ATP3, ATP4, GLO4, YAT1, MTM1, FMP46, PUF3, YML6, CYC3, COX11, CIT3, CYC2, COX14, COX13, CIT2, COX10, YNL208W, GEP3, COA4, COA3, TOM7, TOM6, MRX4, MNP1, MRX6, COQ5, COQ4, ERP6, NAT2, FMC1, MRH4, COX23, ZIP2, MGE1, SWS2, SFA1, APJ1, MTO1, HSC82, QCR7, QCR6, MSS116, MRPL8, CPT1, SYG1, AI5_ALPHA, HSP10, EXO5, LEU5, MRPL9, QCR9, OXA1, YPT1, QCR8, GPX1, IML2, NUM1, HSP26, FMP10, MOD5, SNQ2, DRE2, FUN14, SPG1, BXI1, TMA10, RSM22, MNE1, ESBP6, JID1, YLH47, NFS1, BMS1, RSM28, YAH1, CCL1, RSM27, TIS11, RSM10, YDR514C, JAC1, OXR1, SPF1, YJL043W, RSM18, CCM1, DGR1, RSM19, FUN30, ACS1* |
| GO:0005730 | nucleolus | *SNR54, NSR1, RRP7, SNR55, SNR11, PXR1, RPC19, FHL1, RRN11, SNR18, RPA12, KRI1, NPL3, RPA14, SNR57, RRP9, SNR13, SNR128, RPS9A, SNR5, RRP14, FOB1, RRP12, BUD23, CGR1, ULP1, UTP15, SNR44, SNR85, SNR86, SNR84, SNR82, IMP4, CIC1, SNR47, SNR45, SIS1, ULS1, SRP40, SUB1, MOD5, SNR17A, NHP2, LCP5, SNR191, NOP4, SNR190, SNR30, SNR31, LRP1, NOP1, NIP7, SNU13, RPB10, RPA34, RPF2, SNR37, RRN6, SNR34, RRN7, SNR35, EMG1, TGS1, BMS1, RRS1, GRC3, TRM112, MRT4, UTP5, NOP15, NOP16, SNR9, IFH1, PNO1, SNR8, SNR60, CTK1, NOG2, DIM1, SPB4, TMA23, SPB1, ENP1, EAF6, SNR67, SNR68, RCL1, MRH4, ESF1, DBP8, BFR2, NOP53, NOP10, DBP3* |
| GO:0005743 | mitochondrial inner membrane | *MRS2, MGE1, HSP60, PHB1, YEA6, SSC1, YME1, QCR10, QCR7, QCR6, MTG2, AEP3, LEU5, OXA1, QCR9, QCR8, MIR1, IMP2, HEM25, MSS51, MRX21, ZIM17, OMS1, UPS3, UPS1, COX15, ATP23, ODC1, ATP3, ATP4, YMC2, BI2, YAT1, JID1, MTM1, MRX10, YLH47, COX7, INA22, ICT1, MIC12, ATP17, TIM13, COX11, MIC10, CYC3, CYC2, ATP15, COX1, COX14, TIM12, COX13, TIM17, ATP18, GEP3, COA4, COA3, CRC1, PET100, TIM9, JAC1, COI1, COQ5, COQ4, MIC27, GTF1, TIM22, TIM23* |
| GO:0005763 | mitochondrial small ribosomal subunit | *MRPS17, RSM10, MRPS18, MRPS16, 15S_RRNA, SWS2, RSM22, MRP10, MRP17, RSM28, RSM18, RSM27, RSM19* |
| GO:0005758 | mitochondrial intermembrane space | *COX19, HSP60, MPM1, ATP23, COX17, SSC1, HYR1, QCR6, TIM13, COX11, CYC3, TIM12, TIM17, MIR1, COA4, MIX23, CMC4, MDM35, TIM9, JAC1, NCE103, YNK1, COX23, TIM22, TIM23, DRE2, UPS3, MIX17, UPS1* |
| GO:0005762 | mitochondrial large ribosomal subunit | *MNP1, MRPL19, MRPL27, MRPL28, MRP20, IMG1, IMG2, MRPL24, MRPL13, MRPL44, MRPL33, MRPL22, MRPL31, 21S_RRNA, MRPL8, MRX14, YML6, MRPL9, MHR1* |
| GO:0031429 | box H/ACA snoRNP complex | *SNR191, SNR11, SNR44, SNR85, SNR30, SNR86, SNR31, SNR9, SNR84, SNR8, SNR82, CIC1, SNR37, SNR34, SNR35, NHP2, SNR5, NOP10* |
| GO:0019005 | SCF ubiquitin ligase complex | *CDC34, MET30, DAS1, RAD7, UFO1, CKS1, UCC1, SKP2, SKP1* |
| GO:0000981 | RNA polymerase II transcription factor activity, sequence-specific DNA binding | *GZF3, MAC1, YAP5, YAP6, HAL9, TOD6, TBS1, CUP2, UPC2, FKH2, PZF1, ARG80, RLM1, ADR1, SMP1, SFL1, RGM1, ASG1, STP4, STP3, STP1, MOT3, HAC1, SUT2, RTG1, NRG1, GLN3, MSN1, MSN2, COM2, TDA9, YRM1, DAL81, YHR054C, RSC3, VHR1, DAL80* |
| GO:0000978 | RNA polymerase II core promoter proximal region sequence-specific DNA binding | *GZF3, MAC1, RPN4, FHL1, INO4, TOD6, INO2, CUP2, UPC2, MGA1, FKH2, PZF1, ARG80, HSF1, RLM1, ADR1, SMP1, SFL1, RGM1, STP4, STP3, HMS2, STP1, MOT3, SKN7, RTG1, NRG1, GLN3, MSN1, FLO8, TDA9, YHR054C, RSC3, DAL80* |
| GO:0043565 | sequence-specific DNA binding | *PHD1, GZF3, SOK2, YAP6, FHL1, HAL9, TOD6, TBS1, DOT6, MGA1, FKH2, HSF1, RLM1, ECM23, ADR1, SMP1, SFL1, CSE4, ASG1, STP4, STP3, HMS2, SKN7, SUT2, GLN3, IXR1, MSN1, TDA9, TYE7, DAL82, RSC3, DAL80* |
| GO:0051082 | unfolded protein binding | *MGE1, HSP82, HSP60, APJ1, SSC1, YAR1, CPR6, HSC82, SSA3, SSA4, KAR2, SSA1, SSA2, HSP10, MDJ1, HSP78, YDJ1, SQT1, JEM1, CCT5, BUD27, HSP104, PET100, PNO1, ATP11, TIM9, SIS1, SHQ1, HSP26, XDJ1, HSP42* |
| GO:0008168 | methyltransferase activity | *PPM1, TRM2, RSM22, NOP1, EFM2, RKM5, TRM44, TRM11, DIM1, SPB1, EFM6, GCD14, HPM1, OPI3, COQ5, SFM1, CDC21, EMG1, MGT1, TGS1, SET2, OMS1, BUD23, TRM112* |
| GO:0003700 | transcription factor activity, sequence-specific DNA binding | *PHD1, MAC1, GZF3, YAP5, HMS2, SOK2, HAC1, SKN7, YAP6, FHL1, CUP2, GLN3, MGA1, COM2, FKH2, TYE7, DAL82, HSF1, DAL80, SMP1, SFL1* |
| GO:0051087 | chaperone binding | *MGE1, HSP60, HSP104, VSB1, HCH1, DJP1, JAC1, APJ1, AHA1, BTN2, CUR1, SIS1, ZIM17, HSP10, XDJ1, YDJ1, HSP78, JEM1* |
| GO:0030559 | rRNA pseudouridylation guide activity | *SNR191, SNR11, SNR44, SNR85, SNR86, SNR31, SNR9, SNR84, SNR8, SNR82, SNR37, SNR34, SNR35, SNR5* |
| GO:0031072 | heat shock protein binding | *SSA3, SSA4, KAR2, SSA1, SSA2, SSC1, APJ1, XDJ1, MDJ1, YDJ1* |
| GO:0051787 | misfolded protein binding | *SIS1, SSA3, SSA4, KAR2, SSA1, SSA2, SSC1, HSP78, JEM1* |
| sce04141 | Protein processing in endoplasmic reticulum | *HSP82, SEC23, UBC7, UBC6, APJ1, HLJ1, SSE1, SSE2, ERO1, HSC82, SHP1, SSA3, SSA4, KAR2, SSA1, SSA2, UFD1, YDJ1, FES1, JEM1, SKP1, SBH2, SFB2, HSP26, UBC4, XDJ1, HSP42* |
| sce00190 | Oxidative phosphorylation | *VMA4, MCO10, COX15, COX17, ATP3, ATP4, BI2, QCR10, BI4, QCR7, QCR6, COX7, ATP17, COX11, COX1, ATP15, COX13, QCR9, QCR8, COX10, ATP18* |
| sce03008 | Ribosome biogenesis in eukaryotes | *UTP15, POP6, NOP4, RRP7, POP8, UTP5, MTR2, NOP1, NOG2, SNU13, IMP4, GSP1, NME1, RCL1, EMG1, RDN5-1, BMS1, SNR17A, NHP2, MEX67, NOP10, RIO1* |
| sce03040 | Spliceosome | *PRP2, ECM2, BUD31, SNU13, LEA1, SNU23, CDC40, YSF3, SNU114, SMX3, SSA3, RDS3, SSA4, SSA1, SSA2, SLU7, PRP46, HSH49, DBP2* |
| sce04213 | Longevity regulating pathway - multiple species | *HSP104, SSA3, SSA4, SSA1, SSA2, RAS2, HST1, HSP78, MSN2, CTA1, ATG5* |

**Table S5 Enrichment table of downregulated genes in *Saccharomyces cerevisiae* BY4742**

| ID | Term | Associated Genes Found |
| --- | --- | --- |
| GO:0006412 | translation | *VAS1, RPL3, RPL22A, RPS0B, RPS0A, RPS15, RPL18A, RPS9B, RPL38, RPS13, RPS12, RPS5, RPL33A, RPL21A, RPL33B, RPS24B, RPS24A, RPL2A, MRF1, RPL17A, RPL17B, RPS8B, SES1, RPL2B, RPS8A, RPL25, RPL29, RPL28, GRS1, RPL20A, RPS11B, RPL16A, RPS11A, RPS31, HTS1, GLN4, RPL43A, RPS1A, RPL31A, RPL43B, GCD7, RPS1B, RPL4A, RPL4B, RPL27A, RPL27B, RPS18B, ILS1, RPS4B, RPS4A, GUS1, MRPL32, RPL42B, TIF11, THS1, RPL26A, HBS1, RPS17B, RPS17A, EFT1, EFT2, GCN2, TYS1, RPL13A, SSB1, RPL13B, RPG1, RPL6A, AIM10, RPS16B, RPL6B, RPL37A, RPS28B, RPS28A, RPL40B, DED81, RPL10, MSW1, TIF3, TIF1, RPS6B, RPS6A, RPL12A, RPL12B, YHR020W, RPL40A, RPS27B, YDR341C, RPL1B, RPS7A, RPL1A, RPS3, RPL9A, DOM34, RLI1, RPL35A, DPS1, RPL23B, RPL11A, CDC60, RPL11B, RPS14B, CRS1, RPL19A, RPS26A, RPL19B, RPS30A, MSF1* |
| GO:0002181 | cytoplasmic translation | *RPP2A, RPS4B, RPP2B, RPL22A, RPS4A, RPS0B, RPL42B, RPS0A, RPL7B, RPS15, RPL7A, RPS25A, RPL18A, RPL26A, RPL38, RPS17B, RPS17A, RPS13, RPS12, SLH1, RPL13A, SSB1, RPL13B, RPP1B, RPL33A, RPP1A, RPL33B, RPL21A, TRM7, RPS24B, RPL6A, RPS24A, RPS16B, RPL6B, RPL2A, RPL17A, RPL17B, RPS8B, RPL2B, RPL37A, RPS8A, RPP0, RPL25, RPS28B, RPL29, RPL28, RPS28A, RPL40B, RPL10, TIF3, RPS6B, RPS6A, RPL24A, RPL12A, RPL12B, RPL20A, RPL40A, RPS27B, RPL1B, RPL16A, RPS7A, RPS11B, RPS11A, RPL1A, RPS3, RPS31, RBG2, RPL9A, RPL35A, RPL11A, RPL23B, RPL43A, RPS1A, RPL11B, RPL31A, RPL43B, RPS1B, RPS14B, RPL8B, RPL4A, RPL19A, RPS26A, RPL19B, RPS30A, RPL4B, RPL27A, RPL27B, RPS18B, RPL8A* |
| GO:0008652 | cellular amino acid biosynthetic process | *BAT1, TRP5, TRP4, MDE1, TRP2, MRI1, LEU3, SER3, LEU1, LEU4, ARG8, LEU9, MET6, LYS12, MET28, ARG4, ILV5, CPA2, PRO1, ILV3, HIS4, ARG3, GLT1, HIS7, ARG1, STR2, LYS1, ARO4, CYS4, CTR86, ARO3, PHA2, HOM2, ARG56, ARO1, LYS9, ACO2, LYS21, MIS1, MET17* |
| GO:0031505 | fungal-type cell wall organization | *BAR1, WSC2, PST1, CRH1, CWP2, CWH43, CRR1, PAU5, HSP150, MYO3, CCW14, PAU7, MYO5, CCW12, UTR2, SKN1, RHO2, SUR7, CNA1, ECM33, SVP26, PAU15, MID2, SUN4, KNH1, MSB2, GAS2, PIR5, CIS3, GAS1, CHS2, SIM1, YPS1, DAN4, GAS5, PIR1, PIR3* |
| GO:0008152 | metabolic process | *MAL12, GSY1, CRH1, URA2, MAL32, CRR1, NTH2, IMA1, SCW11, ADE57, SCW10, PFK2, FAS1, IMA5, FAS2, ADE17, ERG9, PDC5, HIS4, HIS7, IDH2, PDC1, SCW4, ARG56, ARO1, GDB1, SUN4, CTS1, DCD1, HXK2, CDC19, MNS1, SIM1, PYC2, RNR1, ROT2, MIS1* |
| GO:0006886 | intracellular protein transport | *ARF3, SEC26, SSO2, APL5, USO1, APL6, APL3, APL1, APL2, KAP123, MTR10, UBX4, COP1, NMD5, VPS8, BUB2, COG3, APM3, CHC1, DRS2, ERP1, KAP114, ERP2, ERP3, ERP4, GYP1, MSB3, YPT11, YHR022C, SSS1, PEP8, PIR1, PEP5* |
| GO:0071555 | cell wall organization | *WSC2, WSC3, WSC4, SMK1, CRH1, CWH43, HSP150, SCW11, SCW10, CCW12, UTR2, SKN1, FKS1, ECM33, SCW4, FIG2, GPI13, SUN4, KNH1, CTS1, CHS7, GAS2, CIS3, GAS1, ECM13, CHS3, CHS2, SIM1, DSE2, GAS5, PIR1, PIR3* |
| GO:0005975 | carbohydrate metabolic process | *MAL12, CRH1, MAL32, YMR196W, CRR1, GLC3, NTH2, IMA1, SCW11, SCW10, UTR2, PGM2, IMA5, SKN1, PGM1, ARA1, SNF1, MDH2, SCW4, GDB1, SUN4, CTS1, GPH1, HXK2, TAL1, GPD1, YHR210C, PKP2, MNS1, PCL7, SIM1, ROT2* |
| GO:0006897 | endocytosis | *INP53, ARF3, APL3, APL1, LSP1, INP51, VPS1, ARV1, ROY1, YPT7, MYO3, MYO5, SYP1, SUR7, ACT1, VMA3, SCD5, SVP26, APM3, CHC1, DRS2, AGE2, MSB3, VRP1, OSH6, YCK3, KES1, LDB17* |
| GO:0000462 | maturation of SSU-rRNA from tricistronic rRNA transcript (SSU-rRNA, 5.8S rRNA, LSU-rRNA) | *NOP14, RRP3, UTP13, RPS6B, RPS6A, ENP2, RPS1A, RPS1B, RPS24B, RPS24A, RPS14B, RPS27B, RPS16B, RPS8B, RPS11B, RPS8A, RPS11A, RPS9B, TSR2, HRA1, SLX9, RPS13, ECM16* |
| GO:0022625 | cytosolic large ribosomal subunit | *POP5, RPL3, WSC2, TSL1, SSO2, UBP15, SMC2, RPS25A, MSA2, PAU5, AIP1, SUT1, FAS1, RPL38, DBF20, KEL1, ASC1, ARA1, TRI1, CYK3, RME1, ATG1, REV1, PAT1, PHA2, RPS24B, RPS24A, SAK1, CIK1, RPL2A, SES1, RPL2B, RPL25, FAR1, RPL29, PYC2, RPL28, HAL1, TPH3, SRO7, MDE1, GRS1, MYO1, HRP1, FPR2, ADH5, GLG1, PBY1, MYO3, MYO4, MYO5, RPS11B, RPS11A, SAN1, ATG7, MKT1, SNF1, SMD1, KEL3, STR2, HTS1, LAS17, CDC5, MSB1, RPL4A, MSB3, SAM1, RPL4B, YHI9, YNR014W, ARF3, RIB2, YSA1, RAS1, RPL42B, GIC2, GIC1, HBS1, RPS17B, RPS17A, ARG3, APC1, TRM3, TPI1, ARG1, STT4, LYS1, TRM7, LYS9, IMD3, LSG1, RPS16B, TAL1, TCP1, ADK1, RPP0, GAS2, IDS2, CLU1, RPS28B, YIM1, RPS28A, MET18, MET17, RPL40B, RPL10, TRP5, GSY1, TRP4, TSR3, USO1, TRP2, MUK1, RPL40A, MSG5, DDC1, RPS27B, SRP72, RPL1B, SCW10, RPL1A, RPS3, DTD1, MIT1, PMS1, DOM34, RLI1, APE1, MDH2, ARI1, ELP2, ELP3, SRP68, SEY1, ARG56, HOM2, RPS14B, LRG1, RPS26A, DEF1, MSF1, MRD1, TSR2, YDR444W, PPX1, YGK1, ISU2, SMK1, MUM2, WTM1, RPL22A, TDA10, CAB4, NTH2, ADD37, ADE57, BEM2, YOR342C, SRV2, CPA2, RGA1, GRX2, SLH1, RPS5, TPM2, RKI1, CYS4, ENV7, RPL33A, RPL33B, RAV1, RPL21A, PPZ1, DCD1, MSH3, DUG1, KES1, LDB17, SKI8, SKI3, RGD2, MRI1, RPL20A, YBL029W, SDS22, ADP1, SAP190, MET28, YIL001W, PDC5, CSI1, PDC1, GLN4, RPL43A, RPL31A, RPL43B, LSM8, YGR130C, PSA1, MOB2, MCK1, YOR387C, ACO1, CHS3, RNR1, RPS18B, KAP122, MCM7, SHE2, SHE3, KSS1, LSP1, KAP123, YNL234W, ADE12, RPL7B, RPL7A, RPL26A, PFK2, PGM2, ADE17, TPS3, LEU4, PGM1, CNA1, ARO8, DIS3, PHM8, HOS3, ARO4, RPL13A, RPL13B, ARO3, YNR061C, FDC1, ARO1, KAP114, RPL6A, AIM10, RPL6B, VRP1, DBF2, RPL37A, MCM3, MCM5, MCM2, EIS1, DED81, TIF3, APL5, TIF1, ARP5, RPL24A, RPL12A, RPL12B, PXP1, TMA17, PXP2, ROY1, MET3, VPS52, PAC1, KAR4, OAF3, ABP1, MET5, MET6, RPL9A, ACT1, INP2, TCA17, SVP26, MSN4, RPL35A, PSE1, RPL11A, RPL23B, CDC60, RPL11B, GDB1, MRL1, CDC53, TRX1, RPL8B, OSH6, YCK3, NCL1, RPL8A, ACS2, SEC7, SEC65, VAS1, SEC6, FAB1, UBX4, JHD2, RPS0B, RPS0A, BTS1, RPS15, YOR296W, CDC20, RPL18A, CDC25, FRD1, ECM29, RPS13, AAP1, RPS12, EXO70, CLA4, MDM36, PEX34, FAA2, SPC42, DUO1, PRE4, XRN1, RPL17A, RPL17B, TKL1, RKR1, SEC53, MSS1, ADY3, CLB2, CLB1, TWF1, NMD4, VPS1, ATX1, NMD5, RPL16A, SWI6, SWI5, YIL108W, RPS31, AAT2, SED4, TFC4, GIP4, RPS1A, RPS1B, GYP1, NCP1, CDC14, RPL27A, RPL27B, REH1, CDC19, YBP2, ILS1, PUP1, YBP1, SEC26, MTR10, TIF11, CTT1, FYV8, CCR4, EFT1, EFT2, SIP3, SEC10, YJL070C, SDD3, SEC18, SSB1, DUS3, CSS1, TMT1, PEP8, SEG1, PCL7, PCL2, SHM2, DCR2, CBS2, KRE29, PTP3, RAD5, LIF1, YHR020W, QRI1, AMN1, APT1, YKR075C, RBG2, CCT4, SYP1, CAR2, DCS2, GAD1, CIA2, TSA1, CIA1, CRS1, RPL19A, RPL19B, DBP5, LOT6, RPP2A, RPP2B, NCW1, RPS9B, VMS1, LMO1, HIS4, HIS7, ARP10, HEK2, RPP1B, ERG10, RPP1A, AGE2, PRM3, CMK1, RPS8B, RPS8A, GPD1, RTN2, URA2, URA1, COP1, FMP48, YHB1, YJR098C, PRO1, HEM3, HEM2, CTR86, PNG1, FUS3, ERP1, RRI1, FUS2, ERP2, ERP3, ERP4, PBP2, PAN3, NAT1, NAT3, FMP30, UBA1, PAN5, TDH2, YPQ1, INP53, STM1, RPS4B, SPE4, RPS4A, EPO1, GUS1, INP51, SAH1, THS1, HCM1, YOR062C, GCN2, TYS1, PEX1, RPG1, DNM1, TTI1, AFG1, GPH1, GND1, RSP5, LCB2, RPS6B, RPS6A, YMR196W, GLC3, SDP1, UGP1, SER3, YDR341C, RPS7A, TYW1, BUB2, YGR117C, ICL1, ATE1, MLP2, DPS1, ADE6, ADE2, ADE1, MVD1, RPS30A, BUD14, HER1* |
| GO:0005840 | ribosome | *NUR1, YVC1, WSC2, WSC3, SSO2, WSC4, MME1, FAB1, EMC10, FRT2, CWH43, IFA38, VTH1, MCR1, PHS1, PAU5, CDC25, CMR2, ERG1, BUD9, BUD8, ERG2, ERG5, ERG9, MTC7, SUR7, YPR027C, YPR003C, ATG1, ALG9, COG6, SEC59, PHO90, COG3, SHH4, ALG2, YSC83, ALG3, PEX32, FAA2, PEX34, TNA1, PIC2, YOR186W, NFT1, YCR024C-B, FIT1, SRO7, YAL065C, ADY3, YMC1, FPR2, GAA1, YLL053C, VPS1, ERI1, NUP85, NUP84, VPS8, RCE1, VPS15, ATG7, RHO2, SNF1, SED4, VAC8, BST1, EMP70, CDC10, SAM3, NCP1, MSB2, YHL017W, HXT17, TVP18, FRE8, DER1, BAP2, ORM1, SEC26, HEH2, STE2, STE3, RAS1, YPK9, HOL1, GPI10, ARV1, GPA1, PNS1, ASG7, SIP3, SMF3, CSC1, NCA2, SEC13, SMF1, OM14, ASI1, STT4, NFG1, VHT1, ITR1, STD1, AGA1, YAL064W-B, YDR524C-B, YGR026W, PTM1, PFA3, HMG1, GPI13, CSS1, YRO2, GAS2, SSS1, GAS1, SEG1, RCF2, PMP1, GAS5, PEP5, ODC2, HFL1, USO1, TCB3, MRX11, VMA16, YLR111W, SRP72, GPC1, UTR2, YHR130C, MCH1, LAG1, SCD5, PMA1, AGC1, PMA2, SEY1, FIG1, FIG2, FIG4, PER1, ERG28, YHR022C, TCB2, CHO1, TCB1, DAN4, DBP5, MAL11, HEM15, HEM14, NUP188, PUT2, NCW1, VPH1, VPH2, YNR066C, ERG24, YBT1, GGC1, SHR3, YCF1, VMS1, IRC9, VMA3, PRM2, VMA5, PRM1, VMA7, FLO1, YDL199C, ALG12, ENV7, IST2, YER039C-A, HNM1, FLO5, PRM4, RAV1, PRM3, MID2, PRM6, YNR065C, PCP1, THI72, ALO1, AQY3, AQY1, AQY2, PMT4, PMT3, PMT6, PMT5, TIP20, KES1, VMR1, YAR066W, TGL1, RTN2, RTN1, YLR342W-A, SPO75, AVT5, CWP2, URA2, YOL107W, ATP1, SPC2, EMA35, SPC3, AVT1, HXT2, MFA1, YNL058C, DPP1, HXT3, MFA2, HXT1, HXT6, HXT7, HXT4, COP1, QDR2, ADP1, UIP4, NUP159, OST4, MRX9, OST6, GEP7, PRM10, NIC96, MRH1, AUS1, CHC1, COQ8, GWT1, FUS1, DRS2, ERP1, ZRG17, ERP2, ERP3, ERP4, COQ1, YJL132W, CHS7, FMP33, CHS6, FMP30, SSY5, CHS3, CHS2, DSE2, YPQ1, MIC60, INP53, INP54, OAC1, PST1, NUP145, SHE3, CRH1, ERS1, ATR2, INP51, EMA17, GPI1, YPT7, PSD1, YFL051C, CCW14, CCW12, PFK2, MMR1, STE14, NUP133, VAN1, BRL1, PUG1, PEX1, YNR061C, TAT2, DNM1, COS6, COS3, MNS1, YPS1, ZRT2, AIM14, SAG1, SSM4, EIS1, WBP1, LCB2, ILM1, APL5, APL6, APL3, YER053C-A, NUP120, APL1, APL2, MAK11, MAL31, ROY1, VPS52, FLO11, TOS7, KAR5, SKN1, YCR101C, FKS1, INP1, ECM33, INP2, SVP26, ALE1, APM3, MRL1, TRX1, DIE2, UGO1, EXP1, YOS9, YPT11, OSH6, PDR12, YCK3, STE23* |
| GO:0046354 | ribonucleoprotein complex | *FAR7, SEC7, VAS1, RPL3, PPH22, UBP15, RPS0B, UBP11, VTH1, RPS0A, RPS15, RPS25A, RPL18A, CDC25, CMR2, FRD1, FAS1, RPL38, FAS2, ASC1, RPS13, RPS12, ARA1, ATG1, RPS24B, RPS24A, SAK1, RPL2A, XRN1, RPL17A, SES1, RPL17B, RPL2B, RPL25, TKL1, RPL29, PYC2, RPL28, SEC53, RKR1, MSS1, SRO7, MDE1, VPS1, ATX1, NMD5, HSP150, RPL16A, RPS11B, RPS11A, THI3, RPS31, ATG7, MKT1, RHO2, AAT2, SMD1, HTS1, VAC8, RPS1A, YNCJ0002C, GCD7, MMF1, RPS1B, RPL4A, NCP1, YHL017W, MSB3, SAM1, RPL4B, HXK2, SAM2, RPL27A, RPL27B, SGF73, ARF3, ILS1, MET13, PUP1, YSA1, MET10, RPL42B, SAP4, TRS130, GPA1, HBS1, DAK2, RPS17B, RPS17A, EFT1, EFT2, ARG4, TPI1, ARG3, YJL070C, ARG1, YNCD0024C, SEC18, SSB1, PTM1, TMT1, LSG1, RPS16B, ADK1, PEP8, RPP1, AZF1, RPS28B, GLY1, RPS28A, MET18, PEP5, HIR2, RPL40B, RPL10, TRP4, MUK1, RPL40A, YNCL0009C, RPS27B, RPL1B, RPL1A, RPS3, CAR2, MDH2, GAD1, PMA1, CIA2, TSA1, CIA1, RPS14B, CRS1, RPS26A, RPL19A, RPL19B, MIS1, YNCD0005C, LOT6, RPP2B, RPL22A, YNR066C, RPS9B, ADE57, VMS1, CPA2, HIS4, GRX2, SLH1, RPS5, GRX4, ERG10, RPL33A, RPL21A, YNCE0021C, RPL33B, RAV1, AGE2, YNR065C, RPS8B, GPD1, RPS8A, AQY2, TIP20, KES1, CWP2, URA2, ATP1, EMA35, RPL20A, YHB1, YNCC0010C, RKM3, SAP190, PRO1, HEM2, PDC5, PDC1, CTR86, GLN4, RPL43A, CHC1, RPL31A, RPL43B, YTH1, PNG1, RIX1, CHS6, YOR387C, ACO1, ACO2, RPS18B, TDH2, SHE2, YML003W, RPS4B, SPE4, YNCL0034W, RPS4A, GUS1, RPL7B, SAH1, RPL7A, THI20, YPT7, TRS85, LEU1, RPL26A, ADE13, PGM2, ADE17, PGM1, YNCK0005C, GCN2, RFC4, NUP133, RPL13A, RPL13B, PEX1, KAP114, DNM1, RPL6A, RPL6B, GND1, SYF1, RPL37A, YNCO0019W, DED81, APL6, RPS6B, RPL24A, RPS6A, RPL12A, RPL12B, APL2, PXP2, SER3, YDR341C, RPS7A, VPS52, KAR4, RPL9A, TCA17, MSN4, RPL35A, DPS1, RPL11A, RPL23B, RPL11B, TRX1, RPL8B, MVD1, SUF17, RPS30A, OSH6, STE23, BET2, RPL8A, ACS2* |
| GO:0022627 | cytosolic small ribosomal subunit | *FAR7, SSO2, UBP15, EMC10, FRT2, NCW1, CWH43, VPH2, IFA38, ERG24, YBT1, SHR3, PHS1, ERG1, ERG2, ERG5, ERG6, SIL1, VMS1, ERG9, YPR027C, YPR003C, ALG9, SEC59, ALG2, ALG3, ALG12, HNM1, FAA2, YNR065C, AQY1, AQY2, PMT4, CIS3, PMT3, PMT6, PMT5, TIP20, RTN2, RTN1, SPC2, SPC3, GAA1, FPR2, MFA1, YNL058C, MFA2, ERI1, EUG1, ADP1, RCE1, PRY2, UIP4, PRY1, OST4, MRX9, OST6, SED4, PRM10, MRH1, GWT1, FUS1, DRS2, ERP1, ERP2, ZRG17, CTS1, ERP3, BST1, ERP4, SAM3, NCP1, CHS7, CHS3, SIM1, ROT2, YPQ1, FRE8, DER1, BAP2, ORM1, INP54, SHE3, STE3, GPI1, GPI10, ARV1, PSD1, STE14, SIP3, SMF3, CSC1, SEC13, VAN1, YAL064W-B, AGA2, BRL1, YDR524C-B, YGR026W, TAT2, PFA3, HMG1, GPI13, CSS2, YRO2, SSS1, MNS1, PEP4, ZRT2, GAS5, YIM1, AIM14, SSM4, HFL1, WBP1, LCB2, ILM1, USO1, TCB3, YER053C-A, ROY1, SRP72, GPC1, SCW10, YBR096W, TYW1, KAR5, LAG1, ECM33, SVP26, ALE1, SEY1, PER1, DIE2, ERG28, EXP1, YOS9, YPT11, OSH6, TCB2, CHO1, TCB1, GTB1* |
| GO:0005737 | cytoplasm | *MAL12, YVC1, WSC2, MAL11, WSC3, SSO2, CWH43, CDC25, BEM2, BUD9, FRD1, BUD8, EXO70, SUR7, LMO1, PRM1, PHO90, FLO1, IST2, MID2, PRM6, THI72, AQY3, AQY1, AQY2, CIS3, YAR066W, SRO7, RGD2, SPO75, AVT1, HXT2, MFA1, HXT3, MFA2, YLL053C, HXT1, HXT6, HXT7, HXT4, MYO3, MYO5, QDR2, RHO2, MRH1, AUS1, FUS1, DRS2, MSB1, SAM3, NCP1, MSB2, MSB3, HXT17, SSY5, CHS3, CDC19, CHS2, TDH2, FRE8, BAP2, PST1, STE2, STE3, YML003W, ERS1, LSP1, RAS1, GIC2, YFL051C, GPA1, SST2, PNS1, ADE17, ASG7, AIM25, SIP3, CAP1, SMF3, CSC1, EFR3, SMF1, TPI1, STT4, VHT1, ITR1, STD1, SSB1, PUG1, YDR524C-B, YGR026W, TAT2, IMD3, COS3, YRO2, TCP1, GAS2, GAS1, SEG1, PMP1, SHM2, YPS1, ZRT2, GAS5, AIM14, MET17, RSP5, EIS1, TCB3, TIF1, APL3, APL1, APL2, MAL32, UGP1, FLO11, TOS7, ABP1, SKN1, MET6, SYP1, FKS1, ECM33, PMA1, PMA2, HOM2, YHR022C, OSH6, TCB2, PDR12, TCB1, YCK3, DAN4* |
| GO:0005576 | extracellular region | *POP5, SEC65, RPL3, RPP2A, RPP2B, RPL22A, RPS0B, RPS0A, RPS15, RPS25A, RPL18A, RPS9B, RPL38, ASC1, RPS13, RPS12, RPS5, RPP1B, RPP1A, RPL33A, RPL21A, RPL33B, RPS24B, RPS24A, RPL2A, RPL17A, RPL17B, RPS8B, RPL2B, RPS8A, RPL25, RPL29, RPL28, ECM16, MRPL17, RPL20A, RPS11B, RPL16A, RPS11A, RPS31, SMD1, NOP14, MRPL25, RPL43A, RPS1A, RPL31A, RPL43B, RPS1B, PBP2, LSM8, RPL4A, RPL4B, RPL27A, RPL27B, RPS18B, RPS4B, RPS4A, MRPL32, RPL42B, RPL7B, RPL7A, RPL26A, RPS17B, RPS17A, EFT1, EFT2, UTP14, UTP13, RPL13A, RPL13B, RPL6A, RPS16B, RPL6B, RPL37A, RPP0, RPS28B, RPS28A, RPL40B, RPL10, RPS6B, RPL24A, RPS6A, RPL12A, RPL12B, SNU56, RPL40A, RPS27B, SRP72, RPL1B, RPS7A, RPL1A, RPS3, RPL9A, NOP56, RPL35A, SRP68, RPL23B, RPL11A, RPL11B, RPS14B, RPL8B, RPL19A, RPS26A, RPL19B, RPS30A, MRD1, RPL8A, PRP38* |
| GO:0005886 | plasma membrane | *RPL3, RPP2A, RPS4B, RPP2B, RPL22A, RPS4A, MRPL32, RPS0B, RPL42B, RPS0A, RPL7B, RPS15, RPL7A, RPS25A, RPL18A, RPS9B, RPL26A, RPL38, RPS17B, VMS1, RPS17A, RPS13, ASC1, RPS12, RPS5, PPE1, RPL13A, RPL13B, RPP1B, RPL33A, RPP1A, RPL33B, RPL21A, RPS24B, RPL6A, RPS24A, RPS16B, RPL6B, RPL2A, RPL17A, RPL17B, RPS8B, RPL2B, RPL37A, RPS8A, RPP0, RPL25, RPS28B, RPL29, RPL28, RPS28A, RPL40B, RPL10, RPS6B, MRPL17, RPS6A, RPL24A, RPL12A, RPL12B, RPL20A, RPL40A, RPS27B, RPL1B, RPL16A, RPS7A, RPS11B, RPS11A, RPL1A, RPS3, RPS31, RPL9A, RPL35A, MRPL25, RPL11A, RPL23B, RPL43A, RPS1A, RPL11B, RPL31A, RPL43B, RPS1B, RPS14B, RPL8B, RPL4A, RPL19A, RPS26A, RPL19B, RPS30A, RPL4B, RPL27A, RPL27B, RPS18B, RPL8A* |
| GO:0009277 | fungal-type cell wall | *ORM1, PUP1, INP54, SHE2, WSC4, SHE3, EMC10, FRT2, CWH43, NCW1, VPH2, IFA38, SHR3, GPI10, ARV1, PHS1, PSD1, CDC25, ERG1, ERG2, ERG5, VMS1, ERG9, SIP3, STE14, SMF3, SEC13, YPR003C, ALG9, SEC59, VAN1, ALG2, ALG3, ALG12, HMG1, GPI13, PRE4, AQY1, AQY2, SSS1, PMT4, PMT3, PMT6, PMT5, MNS1, TIP20, RTN2, SSM4, RTN1, WBP1, ILM1, USO1, TCB3, YER053C-A, SPC2, FPR2, SPC3, GAA1, SRP72, ERI1, KAR5, ADP1, RCE1, LAG1, UIP4, OST4, OST6, SED4, SVP26, ALE1, SEY1, GWT1, ERP1, ERP2, ZRG17, ERP3, ERP4, BST1, PER1, DIE2, ERG28, NCP1, EXP1, YOS9, CHS7, YPT11, OSH6, CHO1, TCB2, TCB1, DER1* |
| GO:0005829 | cytosol | *BAR1, PST1, SSO2, CRH1, LSP1, RAS1, ATR2, HOL1, CCW14, YFL051C, CCW12, PNS1, SUR7, SEC13, EFR3, NFG1, VHT1, PHO90, ITR1, FLO1, AGA1, IST2, YGR026W, HNM1, FLO5, TNA1, LYS9, THI72, CSS1, AQY3, CIS3, SEG1, YPS1, SAG1, FIT1, RTN2, EIS1, RTN1, HXT2, HXT3, MAL31, HXT1, HXT6, HXT7, HXT4, MYO3, QDR2, GPC1, MYO5, SCW11, SCW10, TOS7, MET5, TOS1, RHO2, FKS1, PMA2, AUS1, SEY1, FIG2, DIE2, MSB3, HXT17, TCB2, PDR12, TCB1, CHS3, CHS2, DAN4* |
| GO:0043332 | mating projection tip | *YVC1, WSC2, WSC3, PST1, ERS1, RPP2B, VPH1, HOL1, YNR066C, VTH1, YBT1, YPT7, PHS1, PAU5, CCW12, YCF1, YDR415C, SMF1, PRM1, CLA4, NFG1, ITR1, AGA2, RPP1B, SCW4, YNR061C, PRM4, TNA1, PFA3, PRM6, PAU15, MID2, KNH1, COS6, THI72, COS3, GAS2, CIS3, PEP4, SAG1, DCR2, FPR2, YMC1, AVT1, HXT2, YNL058C, CRR1, FLO11, TOS7, ADP1, MET5, TOS1, PRY2, PRY1, YCR101C, APE1, FIG1, CTS1, MF(ALPHA)2, HXT17, YJL132W, PIR5, YCK3, DSE2* |
| GO:0003735 | structural constituent of ribosome | *RPL3, RPP2A, RPS4B, RPP2B, RPL22A, RPS4A, MRPL32, RPS0B, RPL42B, RPS0A, RPL7B, RPS15, RPL7A, RPS25A, RPL18A, RPS9B, RPL26A, RPL38, RPS17B, RPS17A, RPS13, RPS12, RPS5, PPE1, RPL13A, RPL13B, RPP1B, RPL33A, RPP1A, RPL33B, RPL21A, RPS24B, RPL6A, RPS24A, RPS16B, RPL6B, RPL2A, RPL17A, RPL17B, RPS8B, RPL2B, RPL37A, RPS8A, RPP0, RPL25, RPS28B, RPL29, RPL28, RPS28A, RPL40B, RPL10, RPS6B, MRPL17, RPS6A, RPL24A, RPL12A, RPL12B, RPL20A, RPL40A, RPS27B, RPL1B, RPL16A, RPS7A, RPS11B, RPS11A, RPL1A, RPS3, RPS31, RPL9A, RPL35A, MRPL25, RPL11A, RPL23B, RPL43A, RPS1A, RPL11B, RPL31A, RPL43B, RPS1B, RPS14B, RPL8B, RPL4A, RPL19A, RPS26A, RPL19B, RPS30A, RPL4B, RPL27A, RPL27B, RPS18B, RPL8A* |
| GO:1990825 | sequence-specific mRNA binding | *VAS1, DED81, ILS1, TYS1, SHE2, SHE3, GRS1, HTS1, GLN4, DPS1, CDC60, GUS1, CRS1, THS1, YDR341C, SES1* |
| GO:0004812 | aminoacyl-tRNA ligase activity | *INP53, RIB2, MAL12, INP54, TSL1, MET10, SPE4, INP51, CAB4, COQ11, IMA1, ADE57, ADE13, PFK2, FAS1, FAS2, IMA5, TPS3, LEU4, ADE17, CCR4, LEU9, ERG9, ARG4, ILV3, HIS4, HIS7, ARO8, FAA2, TAT2, ARO1, YIL165C, IMD3, DCD1, YHR210C, RPP1, TKL1, SHM2, GLY1, PYC2, MET17, BAT1, LCB2, GSY1, URA2, MAL32, PXP1, GLC3, TYW1, THI3, DSF1, ARG8, ICL1, MET5, AAT2, CAR2, PDC5, MDH2, STR2, GAD1, IDH2, PDC1, ARI1, ELP3, ARG56, GDB1, HXK2, YHI9, CDC19, NIT1, ROT2, RNR1, LYS21, MIS1, BET2, ACS2* |
| GO:0003824 | catalytic activity | *VAS1, DED81, ILS1, MSW1, GRS1, URA2, LIF1, YHR020W, GUS1, PBY1, ADE12, THS1, YDR341C, ADE57, SAN1, CPA2, TYS1, ARG1, HTS1, GLN4, ADE6, DPS1, CDC60, FAA2, ADE1, AIM10, CRS1, MSF1, SES1, UBA1, PYC2, MIS1, RAD18, ACS2* |
| GO:0016874 | ligase activity | *HEM15, TRP5, MDE1, TRP2, PXP1, PHS1, PSD1, LEU1, ADE13, TYW1, FAS1, THI3, ICL1, ARG4, ILV3, PDC5, HEM2, HIS7, GAD1, PDC1, CYS4, PHA2, FDC1, ARO1, ADE2, MVD1, ACO1, ACO2, GLY1, NIT1, MET17* |
| GO:0016757 | transferase activity, transferring glycosyl groups | *WBP1, GSY1, TRP4, CRH1, GLG1, GPI1, CRR1, APT1, GPI10, GLC3, ERI1, UTR2, ALG9, FKS1, VAN1, ALG2, ALG3, ALG12, MNN5, GDB1, DIE2, GPH1, IDS2, PMT4, PMT3, CHS3, PMT6, PMT5, CHS2* |
| GO:0004553 | hydrolase activity, hydrolyzing O-glycosyl compounds | *VAS1, DED81, ILS1, TYS1, MSW1, GRS1, HTS1, GLN4, DPS1, CDC60, YHR020W, GUS1, AIM10, CRS1, THS1, YDR341C, MSF1, SES1* |
| GO:0016829 | lyase activity | *MAL12, CRH1, SCW4, MAL32, GDB1, SUN4, CTS1, CRR1, NTH2, SCW11, IMA1, SCW10, UTR2, IMA5, MNS1, SIM1, ROT2* |
| GO:0016798 | hydrolase activity, acting on glycosyl bonds | *RPS5, RPS4B, RPS4A, RPL11A, RPL11B, RPL2A, RPS11B, RPL2B, RPS11A, RPS9B, RPL37A, RPL25, RPS18B, EFT1, EFT2, RPL9A* |
| GO:0016831 | carboxy-lyase activity | *CTS1, CRR1, GLC3, SCW11, CRH1, SCW10, UTR2, SCW4, GDB1, SKN1, YMR196W, ROT2* |
| sce03010 | Ribosome | *RPL3, RPP2A, RPS4B, RPP2B, RPL22A, RPS4A, MRPL32, RPS0B, RPL42B, RPS0A, RPL7B, RPS15, RPL7A, RPS25A, RPL18A, RPS9B, RPL26A, RPL38, ICR1, RPS17B, RPS17A, RPS13, RPS12, RPS5, RPL13A, RPL13B, RPP1B, RPL33A, RPP1A, RPL33B, RPL21A, RPS24B, RPL6A, RPS24A, RPS16B, RPL6B, RPL2A, RPL17A, RPL17B, RPS8B, RPL2B, RPL37A, RPS8A, RPP0, RPL25, RPS28B, RPL29, RPL28, RPS28A, RPL40B, RPL10, RPS6B, RPS6A, RPL24A, RPL12A, RPL12B, RPL20A, RPL40A, RPS27B, RPL1B, RPL16A, RPS7A, RPS11B, RPS11A, RPL1A, RPS3, RPS31, RPL9A, RPL35A, RPL11A, RPL23B, RPL43A, RPS1A, RPL11B, RPL31A, RPL43B, RPS1B, RPS14B, RPL8B, RPL4A, RPL19A, RPS26A, RPL19B, RPS30A, RPL4B, RPL27A, RPL27B, RPS18B, RPL8A* |
| sce01110 | Biosynthesis of secondary metabolites | *HEM15, TSL1, HEM14, IFA38, ERG24, TDA10, BTS1, PHS1, NTH2, ERG1, ADE57, ERG2, ERG5, ERG6, IDP1, ERG9, ILV5, ILV3, HIS4, GLT1, HIS7, SHH4, RKI1, CYS4, ERG10, PHA2, ERG13, GPD1, TKL1, TGL5, TGL4, SEC53, DPP1, GLG1, ADH5, THI3, RCE1, LYS12, AAT2, PRO1, HEM3, HEM2, PDC5, STR2, IDH2, PDC1, COQ1, SAM1, HXK2, SAM2, PSA1, ACO1, CDC19, ACO2, PAN5, TDH2, LYS21, RIB5, GUS1, CTT1, PSD1, LEU1, ADE13, PFK2, PGM2, TPS3, LEU4, ADE17, LEU9, PGM1, STE14, ARG4, ARG3, TPI1, ARO8, ARG1, LYS1, ARO4, ARO3, FDC1, ARO1, HMG1, LYS9, IMD3, GPH1, GND1, TAL1, ADK1, SHM2, GLY1, MET17, BAT1, TRP5, GSY1, TRP4, TRP2, MET3, UGP1, GLC3, SER3, ARG8, ICL1, MET6, CAR2, MDH2, GAD1, ALE1, ADE6, HOM2, ARG56, GDB1, ADE2, ADE1, MVD1, CHO1, ACS2* |
| sce01230 | Biosynthesis of amino acids | *LEU1, PFK2, LEU4, LEU9, IDP1, ARG4, ILV5, ILV3, HIS4, TPI1, GLT1, ARG3, HIS7, ARO8, ARG1, RKI1, LYS1, ARO4, CYS4, ARO3, PHA2, ARO1, LYS9, TAL1, TKL1, SHM2, GLY1, PYC2, MET17, BAT1, TRP5, TRP4, TRP2, SER3, ARG8, MET6, LYS12, AAT2, PRO1, STR2, IDH2, HOM2, ARG56, SAM1, SAM2, ACO1, CDC19, ACO2, TDH2, LYS21* |
| sce01100 | Metabolic pathways | *TSL1, FAB1, IFA38, BTS1, PHS1, IMA1, ERG1, ERG2, FAS1, IMA5, FAS2, ERG5, ERG6, IDP1, ERG9, ARA1, GLT1, ALG9, SEC59, SHH4, ALG2, ALG3, FAA2, PHA2, TKL1, PYC2, SEC53, MDE1, GAA1, GLG1, ADH5, ERI1, THI3, AAT2, STR2, ISN1, CTS1, BST1, SAM1, HXK2, SAM2, CDC19, RIB2, MET13, YSA1, MET10, RIB5, CTT1, GPI10, DAK2, ARG4, TPI1, ARG3, ARG1, STT4, LYS1, HMG1, GPI13, LYS9, IMD3, TAL1, ADK1, GLY1, SHM2, MET17, BAT1, TRP5, GSY1, TRP4, TRP2, QRI1, VMA16, APT1, ARG8, LAG1, CAR2, MDH2, NDE1, GAD1, NDE2, PMA1, PMA2, ARG56, HOM2, FIG4, CHO1, PPX1, YGK1, MIS1, MAL12, HEM15, HEM14, PUT2, VPH1, GFA1, ERG24, TDA10, CAB4, NTH2, ADE57, ILV5, CPA2, VMA3, ILV3, VMA5, HIS4, HIS7, VMA7, RKI1, CYS4, ALG12, ERG10, MNN5, ERG13, ALO1, DCD1, PMT4, PMT3, PMT6, PMT5, DUG1, TGL5, TGL4, MRI1, URA2, URA1, ATP1, DSF1, LYS12, OST4, PRO1, HEM3, HEM2, PDC5, OST6, IDH2, PDC1, GLN4, GWT1, YJL132W, PSA1, ACO1, CHS3, ACO2, CHS2, NIT1, ROT2, RNR1, PAN5, TDH2, LYS21, INP53, SPE4, GUS1, INP51, GPI1, ADE12, SAH1, THI20, PSD1, LEU1, ADE13, PFK2, PGM2, LEU4, ADE17, TPS3, PGM1, LEU9, ARO8, PHM8, VAN1, ARO4, ARO3, ARO1, GPH1, GND1, MNS1, WBP1, LCB2, MAL32, MET3, GLC3, UGP1, SER3, PAC1, MET5, ICL1, MET6, FKS1, ALE1, ADE6, GDB1, ADE2, ADE1, DIE2, MVD1, ACS2* |
| sce04011 | MAPK signaling pathway - yeast | *BAR1, RSP5, PTP3, WSC2, WSC3, KSS1, CLB2, CLB1, SWI4, STE2, STE3, MFA1, MFA2, MSG5, CTT1, SDP1, SWI6, BEM2, FLO11, GPA1, TEC1, SST2, STE12, RGA1, FKS1, CLA4, STT4, MSN4, FUS1, FUS3, MID2, DIG2, MSB2, MF(ALPHA)2, GPD1, FAR1, YPS1, MF(ALPHA)1* |
| sce00500 | Starch and sucrose metabolism | *MAL12, FKS1, TSL1, GSY1, MAL32, GDB1, GLG1, GPH1, UGP1, GLC3, NTH2, HXK2, IMA1, PGM2, IMA5, TPS3, PGM1* |
| sce01210 | 2-Oxocarboxylic acid metabolism | *ILV5, AAT2, BAT1, ILV3, ARO8, IDH2, ARG56, HOM2, LEU1, ACO1, ACO2, LEU4, ARG8, LYS21, IDP1, LEU9, LYS12* |
| sce00400 | Phenylalanine, tyrosine and tryptophan biosynthesis | *AAT2, TRP5, ARO8, TRP4, TRP2, ARO4, ARO3, PHA2, ARO1* |
| sce00100 | Steroid biosynthesis | *ERG1, ERG2, TGL5, ERG5, TGL4, ERG6, ERG24, ERG9, TGL1* |
| sce00270 | Cysteine and methionine metabolism | *AAT2, BAT1, ARO8, MDH2, STR2, MDE1, CYS4, MRI1, SPE4, HOM2, SAH1, SAM1, SER3, SAM2, MET6, MET17* |


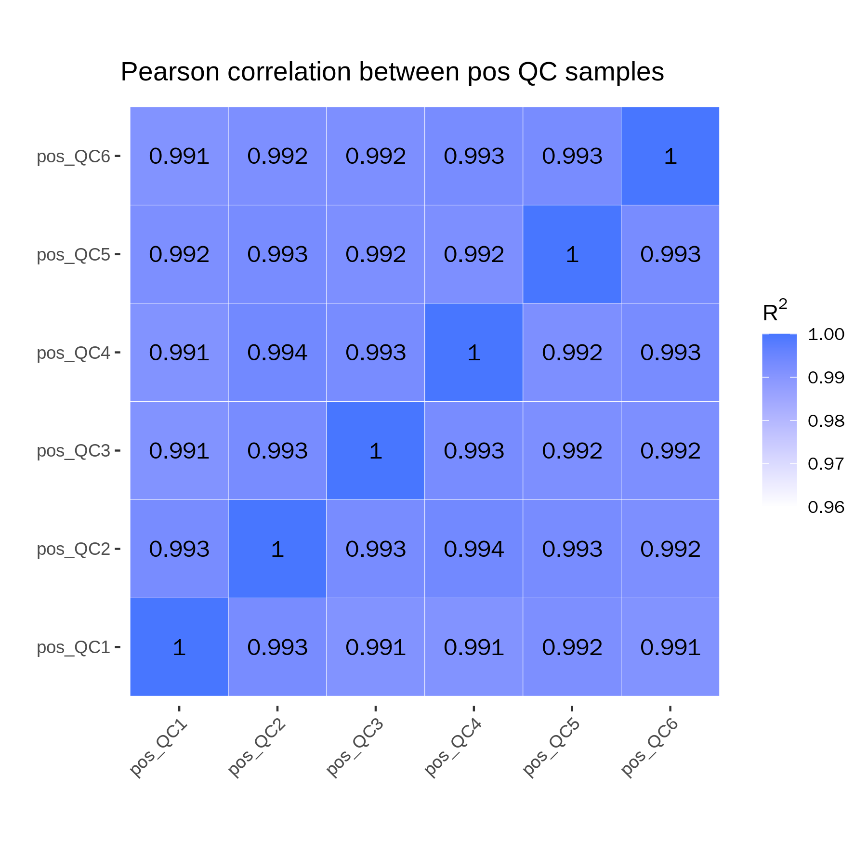


**Fig. S3** Pearson Correlation Analysis of Positively Charged Metabolites among Replicate Samples


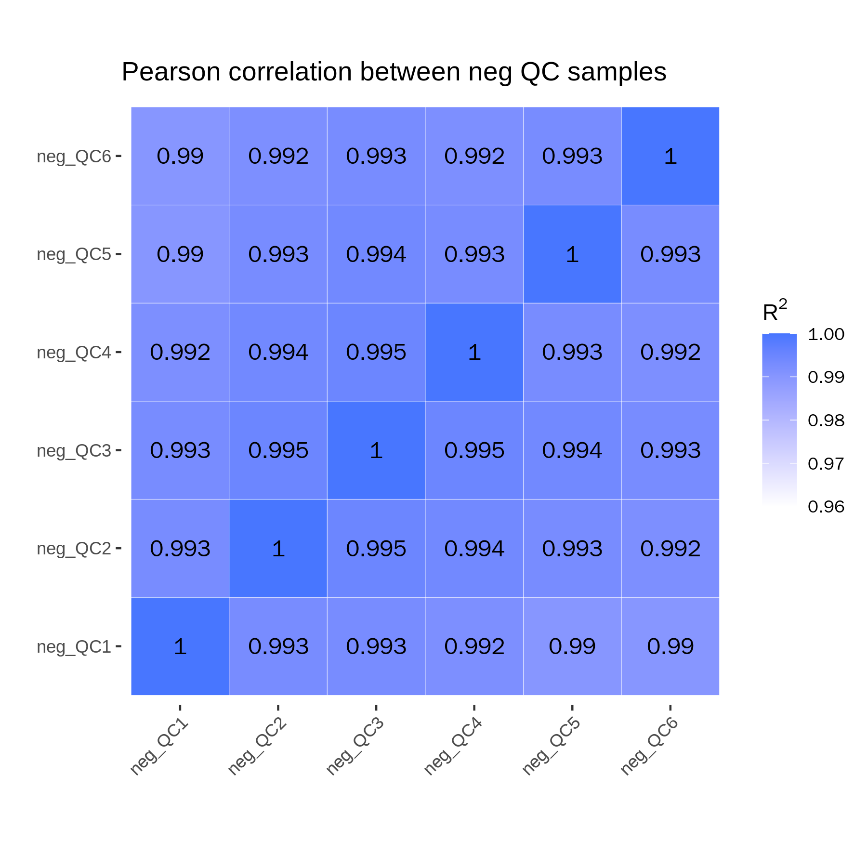


**Fig. S4** Pearson Correlation Analysis of Negatively Charged Metabolites among Replicate Samples


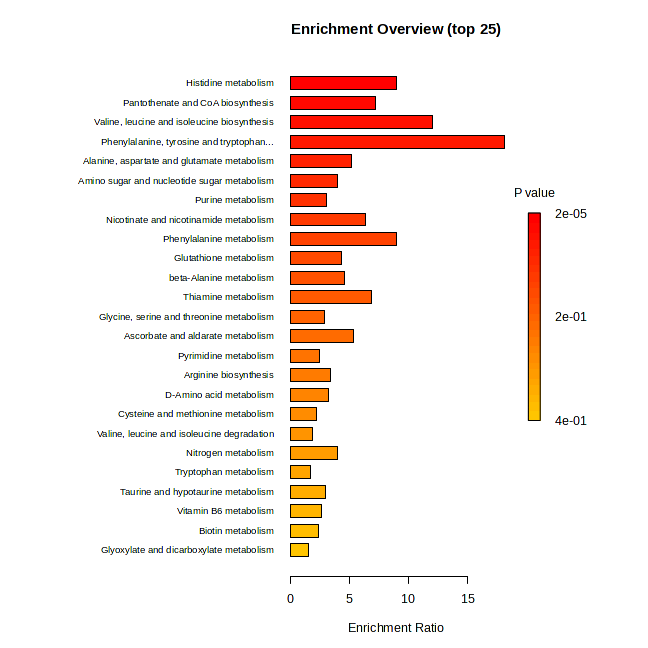


**Fig. S5** Enrichment pathways of upregulated metabolites in *Candida tropicalis* SHC-03


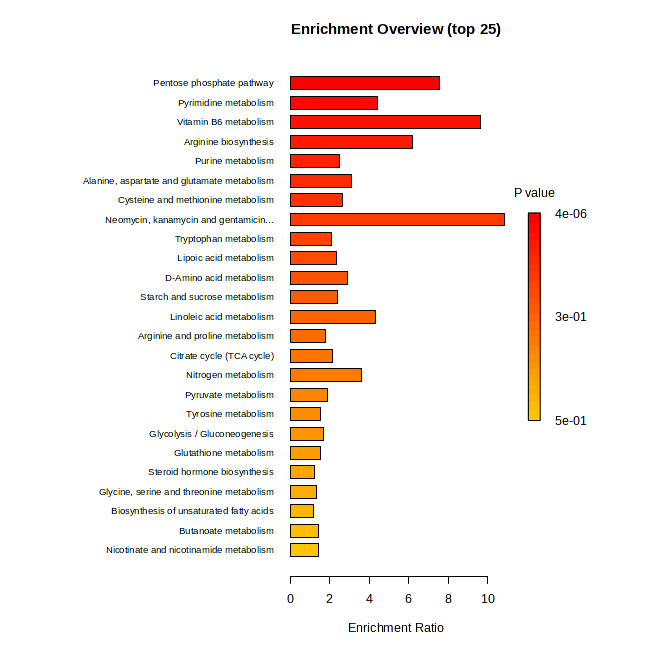


**Fig. S6** Enrichment pathways of downregulated metabolites in *Candida tropicalis* SHC-03


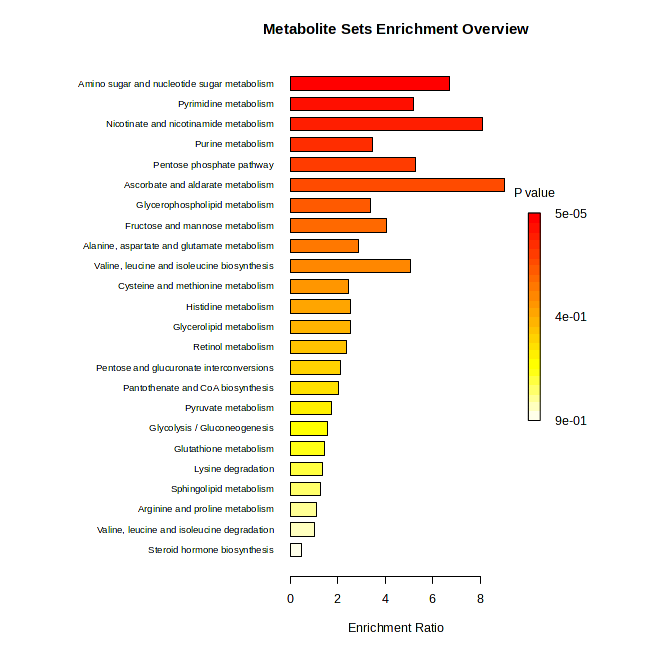


**Fig. S7** Enrichment pathways of upregulated metabolites in *Saccharomyces cerevisiae* BY4742


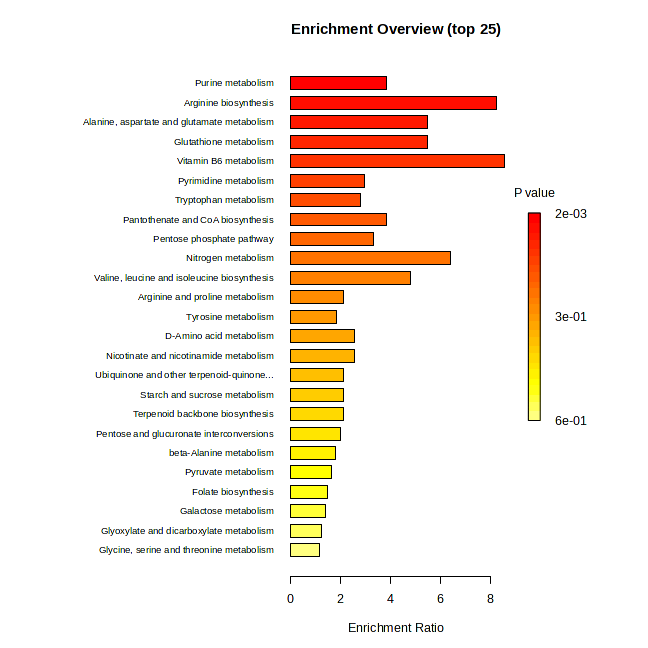


**Fig. S8** Enrichment pathways of downregulated metabolites in *Saccharomyces cerevisiae* BY4742
